# Pleckstrin-2 is essential for erythropoiesis in β-thalassemic mice, reducing apoptosis and enhancing enucleation

**DOI:** 10.1038/s42003-021-02046-9

**Published:** 2021-05-03

**Authors:** Maria Feola, Andrea Zamperone, Daniel Moskop, Huiyong Chen, Carla Casu, Dechen Lama, Julie Di Martino, Mansour Djedaini, Luena Papa, Marc Ruiz Martinez, Tenzin Choesang, Jose Javier Bravo-Cordero, Matthew MacKay, Paul Zumbo, Nathan Brinkman, Charles S. Abrams, Stefano Rivella, Shilpa Hattangadi, Christopher E. Mason, Ronald Hoffman, Peng Ji, Antonia Follenzi, Yelena Z. Ginzburg

**Affiliations:** 1 Tisch Cancer Institute, Icahn School of Medicine at Mount Sinai, New York, NY USA; 2 University of Piemonte Orientale, Amedeo Avogadro, Novara, Italy; 3 Perlmutter Cancer Center, New York University Langone Medical Center, New York, NY USA; 4 Erythropoiesis Laboratory, New York Blood Center, New York, NY USA; 5 Hunan Province Key Laboratory of Basic and Applied Hematology, School of Life Sciences, Central South University, Changsha, China; 6 Children’s Hospital of Philadelphia, Philadelphia, PA USA; 7 Weill Cornell Medical College, New York, NY USA; 8 CSL Behring, Kankakee, IL USA; 9 Perelman Center for Advanced Medicine, University of Pennsylvania School of Medicine, Philadelphia, PA USA; 10 Yale University, New Haven, CT USA; 11 Northwestern University, Chicago, IL USA

**Keywords:** Molecular biology, Anaemia

## Abstract

Erythropoiesis involves complex interrelated molecular signals influencing cell survival, differentiation, and enucleation. Diseases associated with ineffective erythropoiesis, such as β-thalassemias, exhibit erythroid expansion and defective enucleation. Clear mechanistic determinants of what make erythropoiesis effective are lacking. We previously demonstrated that exogenous transferrin ameliorates ineffective erythropoiesis in β-thalassemic mice. In the current work, we utilize transferrin treatment to elucidate a molecular signature of ineffective erythropoiesis in β-thalassemia. We hypothesize that compensatory mechanisms are required in β-thalassemic erythropoiesis to prevent apoptosis and enhance enucleation. We identify pleckstrin-2—a STAT5-dependent lipid binding protein downstream of erythropoietin—as an important regulatory node. We demonstrate that partial loss of pleckstrin-2 leads to worsening ineffective erythropoiesis and pleckstrin-2 knockout leads to embryonic lethality in β-thalassemic mice. In addition, the membrane-associated active form of pleckstrin-2 occurs at an earlier stage during β-thalassemic erythropoiesis. Furthermore, membrane-associated activated pleckstrin-2 decreases cofilin mitochondrial localization in β-thalassemic erythroblasts and pleckstrin-2 knockdown in vitro induces cofilin-mediated apoptosis in β-thalassemic erythroblasts. Lastly, pleckstrin-2 enhances enucleation by interacting with and activating RacGTPases in β-thalassemic erythroblasts. This data elucidates the important compensatory role of pleckstrin-2 in β-thalassemia and provides support for the development of targeted therapeutics in diseases of ineffective erythropoiesis.

## Abstract (web summary)

Maria Feola et al., elucidate the compensatory role of pleckstrin-2 in ineffective erythropoiesis in β-thalassemic mice by reducing cofilin-mediated apoptosis and enhancing enucleation by activating RacGTPases. These findings could support future therapeutic interventions.

## Introduction

Erythropoietin (Epo) is central to erythropoiesis and enables the daily production of billions of red blood cells (RBCs). Epo binds to Epo receptor (EpoR) on erythroid precursors, activating downstream signaling pathways which are responsible for erythroblast proliferation, differentiation, and apoptosis^1^. In addition, terminal erythropoiesis requires erythroblasts to undergo enucleation to form reticulocytes and ultimately mature RBCs^2^, but the precise underlying mechanisms remain uncertain^3^. Several studies indicate that both cytoskeletal re-organization and RacGTPases play an important role in erythroblast enucleation^4–7^.

β-Thalassemia is an inherited disorder associated with ineffective erythropoiesis due to: insufficient or absent β-globin production, resulting in anemia; erythroid expansion in the bone marrow, spleen, and liver, leading to hepatosplenomegaly; and iron overload^8^. Increased erythroblast apoptosis, shortened RBC survival, and defective enucleation all contribute to anemia and have been demonstrated in both human and mice with β-thalassemia^9,10^. Although the excess generation of reactive oxygen species (ROS) has been postulated to cause erythroblast damage in β-thalassemia^11^, to date, the molecular mechanisms underlying ineffective erythropoiesis and defective enucleation in β-thalassemia remain incompletely understood^12^.

Previously, we have shown that exogenous apo-transferrin (apoTf) ameliorates ineffective erythropoiesis and reverses the enucleation defect in β-thalassemic mice^13–15^. Although a complete mechanistic understanding of how apoTf improves ineffective erythropoiesis is unavailable, evaluating apoTf-treated β-thalassemic mice provides an opportunity to explore mechanisms underlying ineffective erythropoiesis and its reversal to better understand regulatory nodes in ineffective erythropoiesis. In this current work, we identify pleckstrin-2 (plek2) as an essential response element in β-thalassemic mice and use plek2 knockout mice to generate plek2 loss in β-thalassemic mice. Several previously published manuscripts provide data on the functional importance of plek2 in erythropoiesis.

Plek2 is a STAT5-dependent lipid-binding protein downstream of Epo, containing a PH domain at both amino- and carboxy-termini. Therefore, like other PH-domain-containing proteins, plek2 is functionally active when present on the membrane^17–20^ including a documented role in membrane trafficking, cellular signaling, and cytoskeletal organization^16^. Plek2 has been shown to interact with cofilin, an actin-binding protein and member of the actin-depolymerizing factor family which regulates actin dynamics^21,22^. Cofilin is also involved in a physiological response to increased ROS^23^ by translocating to the mitochondria and initiating mitochondrial permeability and release of cytochrome *c*, an early step in apoptosis. Binding of plek2 prevents cofilin’s translocation to the mitochondria and consequent apoptosis in response to increased ROS, whereas decreased plek2 liberates cofilin for mitochondrial translocation^23,24^. Furthermore, plek2 supports actin cytoskeleton integrity, is critical for cell proliferation and differentiation, and is required for enucleation^24–27^. Finally, plek2 knockout mice with age develop mild anemia due to ineffective erythropoiesis and increased sensitivity of circulating RBCs to oxidative damage^25^, suggesting that plek2 may have a protective effect on RBC integrity in an oxidative environment without significantly contributing to steady-state hematopoiesis in vivo, at least in normal erythropoiesis.

Our current work interrogates in detail the role of membrane-associated plek2 in terminal erythropoiesis in both physiological and pathological states. We demonstrate: plek2 upregulation and earlier membrane localization in β-thalassemic erythroblasts; an embryonic lethal phenotype in β-thalassemic plek2 knockout mice, decreased survival in β-thalassemic plek2 haplo-insufficient pups, and more ineffective erythropoiesis in β-thalassemic plek2 haplo-insufficient mice that survive to analysis at weaning; increased plek2:cofilin interaction preventing apoptosis despite increased ROS in early stage β-thalassemic erythroblasts; and enhanced plek2-dependent Rac1 activation in β-thalassemic erythroblasts in vivo and in vitro in fetal liver cells (FLCs). Together, our data provide compelling evidence that plek2 loss significantly worsens erythroblast survival and enucleation in β-thalassemic mice, and points to high plek2 membrane localization in early stage β-thalassemic erythroblasts as an important compensatory mechanism preventing further ineffective erythropoiesis in β-thalassemia.

## Results

### RNA sequencing identified increased pleckstrin-2 expression in β-thalassemic pro-erythroblasts as a marker of ineffective erythropoiesis

**Fig. 1:**
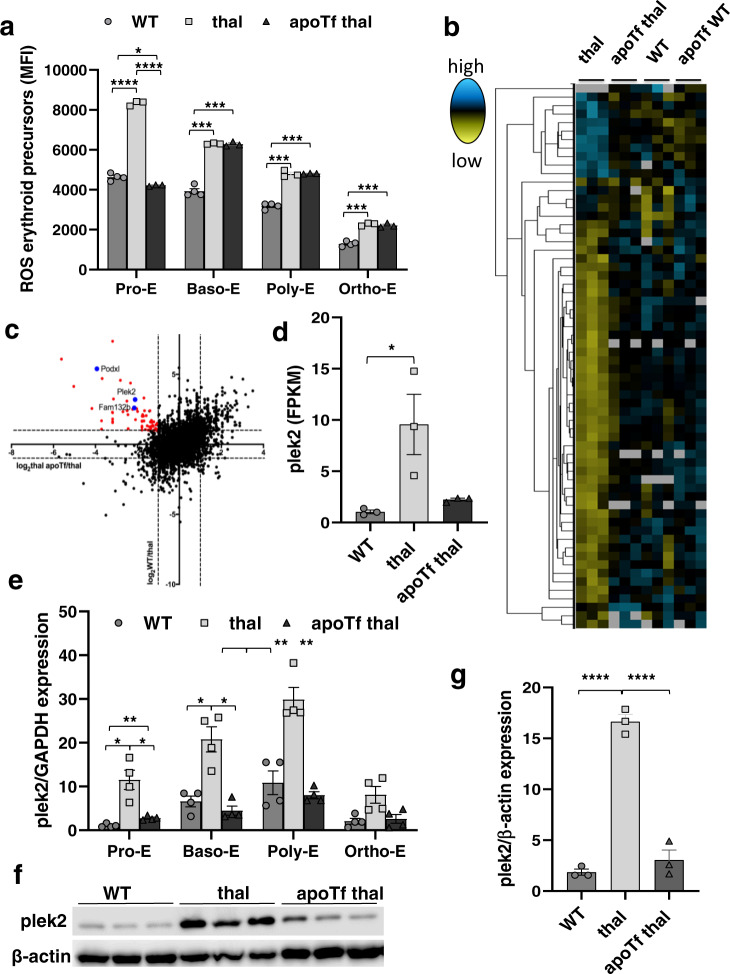
Pleckstrin-2 is an important marker of ineffective erythropoiesis in β-thalassemic erythroblasts. **a** Flow cytometry analysis of MFI of erythroid precursor ROS from WT, β-thalassemic, and apoTf-treated β-thalassemic mice, as identified by CD44 and forward scatter (*n* = 3-4 mice/group), demonstrating highest ROS in Pro-E stage progressively decreasing during erythroid differentiation, increased ROS at all stages in β-thalassemic relative to WT erythroblasts, and decrease in ROS only in Pro-E stages in apoTf-treated β-thalassemic erythroblasts. **b** Heatmap of RNA sequencing analysis showing expression levels (FPKM values) of transcripts that display significant differential expression patterns between β-thalassemic and WT Pro-E but correct after apoTf treatment in β-thalassemic mice. Figure represents 2–3 mice pooled per data point, *n* = 3 data points per group. **c** Log_2_-based fold change between comparisons WT/thal vs. thal apoTf/thal were plotted on *y* vs. *x* axes, respectively. Red data points represent genes whose expression increases in β-thalassemic relative to WT Pro-E and decreases in β-thalassemic Pro-E after apoTf treatment, revealing several genes of interest, e.g., plek2, and known genes that provide validation, e.g., Fam132b and Podxl. **d** Relative expression of plek2 from RNA sequencing analysis demonstrates significantly increased plek2 in β-thalassemic bone marrow Pro-E, which partially corrects after apoTf treatment. **e** Plek2 mRNA expression in sorted bone marrow samples to isolate progressive stages of erythroid differentiation from 2–3 mice pooled per data point, *n* = 4 data points per group WT, β-thalassemic, and apoTf-treated β-thalassemic mice, demonstrating increased plek2 mRNA expression in all four stages of erythropoiesis in β-thalassemic compared to WT erythroblasts, normalized in apoTf-treated β-thalassemic erythroblasts. **f** Plek2 protein expression in CD45^−^ bone marrow cells demonstrate increased plek2 in β-thalassemic mice compared to WT, and normalized in apoTf-treated β-thalassemic mice (*n* = 3 mice/group, MW = plek2 38 kDa, actin 42 kDa). **g** Statistical analysis of the western blot in (**f**) for plek2 quantification. All data are reported as mean ± s.e.m. and *p* < 0.05 is considered statistically significant ((**d** and **g**) ordinary one-way ANOVA; (**a** and **e**) two-way ANOVA)) (*p ≤ 0.05, **p ≤ 0.005, ***p ≤ 0.0005). WT = wild type; apoTf = apo-transferrin; ROS = reactive oxygen species; MFI = mean fluorescence index; Pro-E = pro-erythroblasts; Baso-E = basophilic erythroblasts; Poly-E = polychromatophilic erythroblasts; Ortho-E = orthochromatophilic erythroblasts; plek2 = pleckstrin-2; FPKM = fragments for kilobase millions.

Although erythroblast ROS are increased in β-thalassemic mice^13,28^ and prior reports suggest that ineffective erythropoiesis in β-thalassemia is a consequence of increased ROS^11^, we demonstrate that reversal of ineffective erythropoiesis in apoTf-treated β-thalassemic mice occurs as a consequence of decreased ROS specifically in pro-erythroblasts (Pro-E) stage, despite persistently increased ROS in later erythroblasts (Fig. 1a). Prior work demonstrates that high level of ROS in early stage of terminal erythropoiesis negatively effects enucleation, and the use of ROS scavengers only in early stage wild-type (WT) erythroblasts results in enhanced erythroid enucleation^29^. We and others have previously shown that β-thalassemic mice exhibit an enucleation defect^30,31^, and now demonstrate a significant amelioration of the enucleation defect in ROS scavenger-treated early stage β-thalassemic erythroblasts (Supplementary Fig. 1). Thus, we focus all feasible analyses specifically on early stages of terminal erythropoiesis (i.e., Pro-Es) to evaluate potential compensatory mechanisms in erythroid differentiation and enucleation.

We previously demonstrated fewer β-thalassemic bone marrow Pro-Es relative to WT, normalized in apoTf-treated β-thalassemic mice^3^. In order to identify additional molecular markers of ineffective erythropoiesis in β-thalassemic mice, we performed RNA sequencing analysis of Pro-E sorted from WT, β-thalassemic, and apoTf-treated β-thalassemic mouse bone marrow. The complete datasets generated during the current study are available in GEO (https://www.ncbi.nlm.nih.gov/geo/query/acc.cgi?acc=GSE158839). Of the 14,666 genes identified, we selected genes whose expression differed more than 2-fold between WT and β-thalassemic samples and also returned to normal in apoTf-treated β-thalassemic mice (Fig. 1b). Among the 96 genes that met these criteria, differences in 20 genes were statistically significant (Fig. 1c). In light of recent data about the potential role of plek2 in erythropoiesis^24^, we focused on plek2. Plek2 mRNA expression increased in β-thalassemic relative to WT erythroblasts and returned to normal after apoTf treatment (Fig. 1c, d). In addition, we also evaluated podocalyxin (Podxl), known to be expressed in hematopoietic progenitors^32,33^, and Fam132b (erythroferrone), known to be increased in β-thalassemic erythroblasts and previously reported as partially corrected in apoTf-treated β-thalassemic mouse bone marrow^34^. As expected, Podxl and Fam132b expression mirrored that of plek2, correlating with greater ineffective erythropoiesis in β-thalassemic relative to WT erythroblasts and corrected in apoTf-treated β-thalassemic erythroblasts (Supplementary Fig. 2a, b). These findings validate the dataset. Furthermore, plek2, Podxl, and Fam132b expression were not suppressed by apoTf injection in WT mice (Supplementary Fig. 2c–e), demonstrating that changes in expression in β-thalassemic erythroblasts were not a direct effect of apoTf. To confirm changes in plek2 expression during terminal erythropoiesis, we further sorted bone marrow erythroid precursors, demonstrating increased plek2 mRNA expression (Fig. 1e) and protein concentration (Fig. 1f, g; and full blot Supplementary Fig. 10) in β-thalassemic compared to WT, which corrected in apoTf-treated β-thalassemic bone marrow erythroblasts.

### Loss of pleckstrin-2 in β-thalassemic mice worsens ineffective erythropoiesis and leads to embryonic lethal phenotype in compound mice

**Fig. 2:**
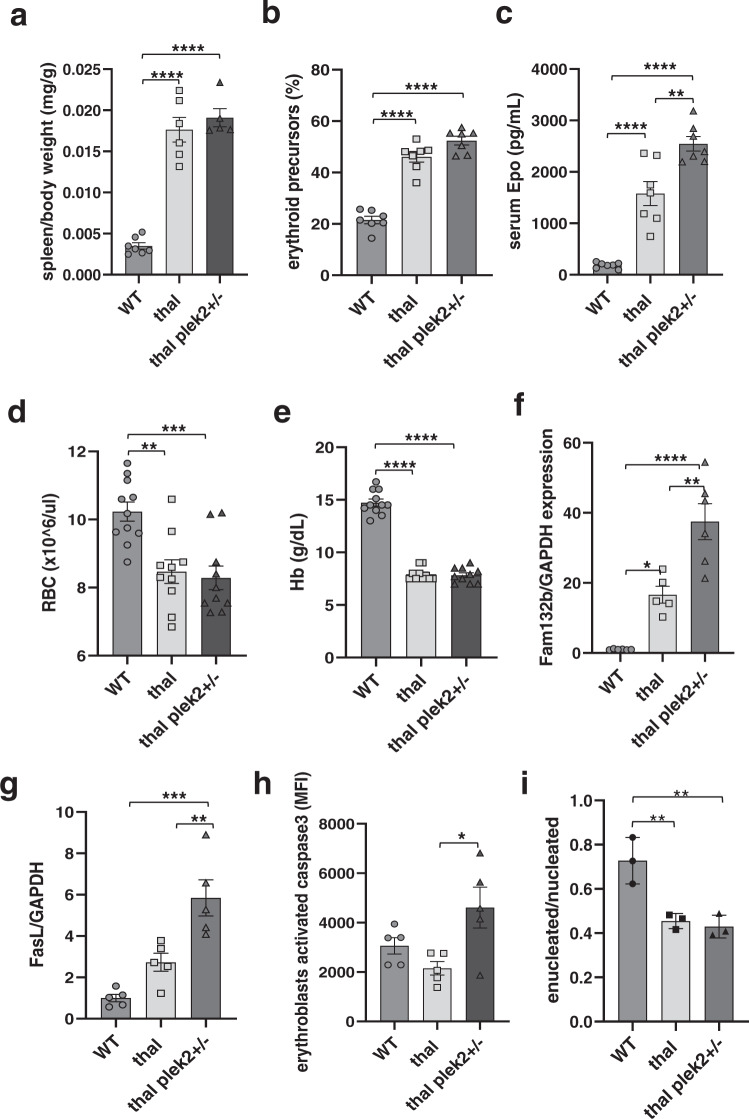
Pleckstrin-2 haplo-insufficiency in β-thalassemic mice results in exacerbation of ineffective erythropoiesis. **a** Splenomegaly observed in β-thalassemic relative to WT mice is not reversed in plek2 haplo-insufficient β-thalassemic (thal plek2+/−) mice (*n* = 5–7 mice/group). **b** Flow cytometry analysis reveals an increased fraction of bone marrow erythroblasts in β-thalassemic relative to WT mice, borderline further increased in thal plek2+/− relative to β-thalassemic mice (*n* = 4–5 mice/group). **c** Serum erythropoietin concentration reveals an increase in β-thalassemic relative to WT mice, further increased in thal plek2+/− relative to β-thalassemic mice (*n* = 4–5 mice/group). **d** Circulating RBC count and **e** hemoglobin were also unaltered in thal plek2+/− relative to β-thalassemic mice (*n* = 10–11 mice/group). **f** Fam132b and **g** FasL mRNA expression in bone marrow CD45^−^ erythroblasts further increased in thal plek2+/− relative to β-thalassemic mice (*n* = 5–6 mice/group). **h** Flow cytometry analysis reveals unchanged caspase 3 activation in erythroblasts from β-thalassemic mice compared to WT, but increased caspase3 activation in thal plek2+/− relative to β-thalassemic mice despite increased serum Epo (*n* = 5 mice/group). **i** Flow cytometry analysis reveals an expected decrease in the fraction of enucleated:nucleated bone marrow erythroblasts (*n* = 3 mice/group). All data are reported as mean ± s.e.m. and *p* < 0.05 was considered statistically significant (one-way ANOVA) (*p ≤ 0.05, **p ≤ 0.005, ***p ≤ 0.0005, ****p ≤ 0.00005). WT = wild type; apoTf = apo-transferrin; RBC = red blood cell; Hb = hemoglobin; Fam132b = erythroferrone.

To elucidate the direct role of plek2 in vivo, we utilize plek2 knockout mice (25) and crossed plek2+/− and β-thalassemic mice to generate β-thalassemic plek2+/− (thal plek2+/−) and β-thalassemic plek2−/− (thal plek2−/−). Of the total 188 pups born, no thal plek2−/− (25% expected) and few thal plek2+/− (4% relative to 25% expected) survived. These survival data prevented the evaluation of thal plek2−/− and demonstrate the significant dependence of β-thalassemic erythropoiesis on plek2 in vivo. Consistently, the thal plek2+/− mice that survived to analysis do not impact splenomegaly (Fig. 2a), exhibit borderline increased fraction of bone marrow erythroblasts (Fig. 2b), but do reveal further increased serum Epo (Fig. 2c). Despite increased serum Epo, RBC count (Fig. 2d) and hemoglobin (Fig. 2e) do not increase relative to β-thalassemic mice. In addition, thal plek2+/− erythroblasts exhibit further increased expression of Fam132b (Fig. 2f), FasL (Fig. 2g), and activated caspase 3 (Fig. 2h), demonstrating more ineffective erythropoiesis resulting from plek2 haplo-insufficiency in β-thalassemic erythroblasts. Lastly, the fraction of enucleated erythroblasts is unchanged in thal plek2+/− relative to thal mice (Fig. 2i). In total, these results demonstrate that the fraction of thal plek2+/− that survive to analysis are the least pathologically affected and that erythroblast apoptosis is increased despite increased Epo in thal plek2+/− mice, together revealing that ineffective erythropoiesis is mitigated by upregulation of plek2 in β-thalassemic erythropoiesis.

To evaluate the severity of embryo lethality, we performed timed matings (thal plek2+/− female X plek2+/− male) and found no thal plek2−/− embryos at E11.5 while thal plek2+/− embryos present in the approximately expected fraction (Supplementary Fig. 3a). Furthermore, we demonstrate that FLCs from thal plek2+/− embryos exhibit persistently defective erythroblast enucleation (Supplementary Fig. 3b) and enhanced erythroblast apoptosis (Supplementary Fig. 3c) relative to plek2+/− embryos. These results strongly suggest that plek2 heterozygosity results in later stage demise in thalassemic embryos, leading to the substantially decreased number of thal plek2+/− weanlings. Taken together, the lethality of plek2 loss in β-thalassemic erythropoiesis is surprising in light of the normal phenotype in plek2−/− mice^25^, with increased erythroblast apoptosis in thal plek2+/− mice (Fig. 2h), which provides new evidence that plek2 serves an important compensatory function in β-thalassemic erythroblasts, confirming a dependence on plek2 in β-thalassemia. We thus next explored the specific mechanism of plek2 function in β-thalassemic erythropoiesis.

### In vitro knockdown of pleckstrin-2 in early stage erythroblasts negatively affects β-thalassemic erythroblast enucleation

**Fig. 3:**
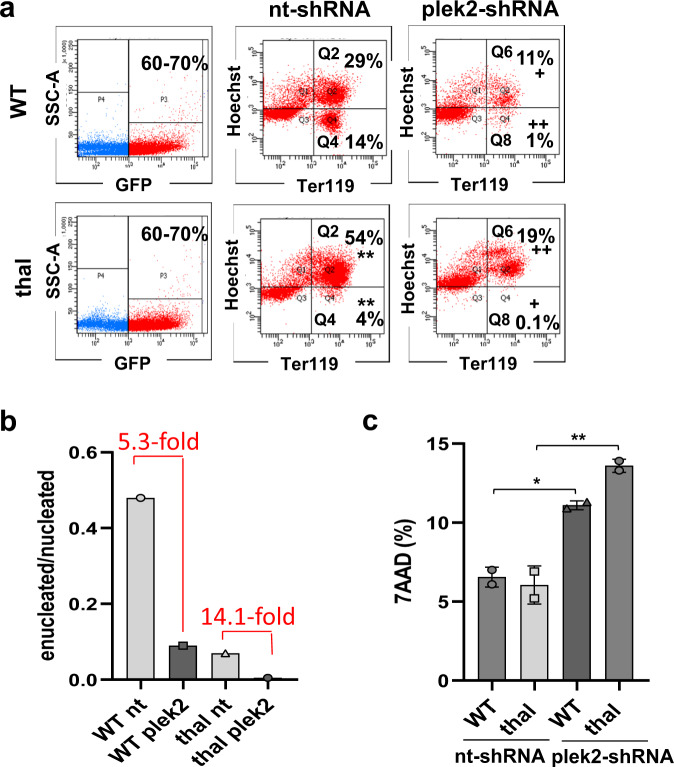
Pleckstrin-2 knockdown in vitro induces cell death and decreases enucleation particularly in fetal liver cells from β-thalassemic embryos. **a** Flow cytometry analysis of nucleated (Q2) and enucleated (Q4) cells after nt-shRNA, and nucleated (Q6) and enucleated (Q8) cells after plek2-shRNA transfection in early stage (CFU-E) erythroblasts derived from Ter119-negative fetal liver cells (FLCs) from E13.5 WT and β-thalassemic embryos, revealing the expected higher degree of proliferation and an enucleation defect in β-thalassemic relative to WT nt-shRNA transfected cells; plek2-shRNA transfection result in a decreased fraction of nucleated (Q6) and enucleated (Q8) cells in WT and β-thalassemic cells. **b** Flow cytometry analysis of (**a**) demonstrates a more severe effect on enucleation in β-thalassemic plek2-shRNA transfected cells compared to WT plek2-shRNA transfected cells. **c** Flow cytometry analysis of cell death using 7-AAD demonstrates no difference between WT and β-thalassemic nt-shRNA transfected FLCs with significant increase in cell death in plek2-shRNA transfected WT and especially β-thalassemic FLCs (from (**a**)). (*n* = 3 mice/group, experiment repeated twice). All data are reported as mean ± s.e.m. and *p* < 0.05 (one-way ANOVA) was considered statistically significant. (**p ≤ 0.005 vs. WT nt-shRNA; ^+^*p* ≤ 0.05 vs. nt-shRNA; ^++^*p* ≤ 0.005 vs. nt-shRNA) WT = wild type; plek2 = pleckstrin-2; MFI = mean fluorescence index; GFP = green fluorescent protein.

We have previously shown that apoTf treatment reverses both elevated serum Epo concentration in β-thalassemic mice^13^ and the enucleation defect in β-thalassemic erythroblasts^31^. Because plek2 expression is STAT-dependent^25^ and its downregulation leads to decreased enucleation in erythroblasts^24^, we hypothesize that increased plek2 prevents an even greater decrease in β-thalassemic erythroblast enucleation. To test this hypothesis and better understand the dependency of β-thalassemic erythropoiesis on plek2, we knocked down plek2 expression in early erythroblasts (CFU-E) using shRNA in FLCs from WT and β-thalassemic embryos. We were able to accomplish a significant decrease in plek2 expression using this method (Supplementary Fig. 4a; and full blot Supplementary Fig. 10). Cultured control non-targeting short hairpin RNA (nt-shRNA) β-thalassemic FLCs demonstrate the expected increased fraction of erythroid precursors (Fig. 3a, Q2) and decreased enucleation (Fig. 3a, Q4) compared to nt-shRNA WT FLCs^31^. Furthermore, plek2 knockdown resulted in significantly fewer nucleated and enucleated cells in both WT and β-thalassemic FLCs (Fig. 3a, Q6 and Q8), demonstrating a 2–3-fold greater negative impact in β-thalassemic relative to WT FLCs (Fig. 3b). In addition, plek2 knockdown increased the fraction of dead cells in both WT and β-thalassemic plek2-shRNA (Fig. 3c). Finally, persistent suppression of enucleation was evident even 72 h after plek2-shRNA transfection in cultured β-thalassemic FLCs but to a degree similar to effect in WT cells (Supplementary Fig. 4b). These results are consistent with previously reported defective enucleation and consequent cell death due to actin cytoskeletal disruption^24^, further confirming the dependence of β-thalassemic erythroblasts on plek2 by preventing worsening of the enucleation defect.

We also repeated this experiment with a delayed knockdown to evaluate the effect of plek2 loss in later stage (Pro-E and Baso-E) erythropoiesis. We also demonstrate a significant decrease in plek2 expression in these experiments (Supplementary Fig. 4c; and full blot Supplementary Fig. 10). Results from nt-shRNA FLCs confirm more erythroid precursors (Supplementary Fig. 4d, Q2) and less enucleation (Supplementary Fig. 4d, Q4) in β-thalassemic relative to WT embryos^35^. Delayed plek2 knockdown in later stage erythroblasts results in a smaller decrease in enucleation (Supplementary Fig. 4d, Q8) but no decrease in nucleated erythroblasts in both WT and β-thalassemic FLCs (Supplementary Fig. 4d, Q6). These results demonstrate that the importance of plek2 in erythroblast enucleation is a consequence of its role especially in early stages of erythropoiesis.

### Pleckstrin-2 membrane localization and activation occurs earlier during β-thalassemic erythroid differentiation

**Fig. 4:**
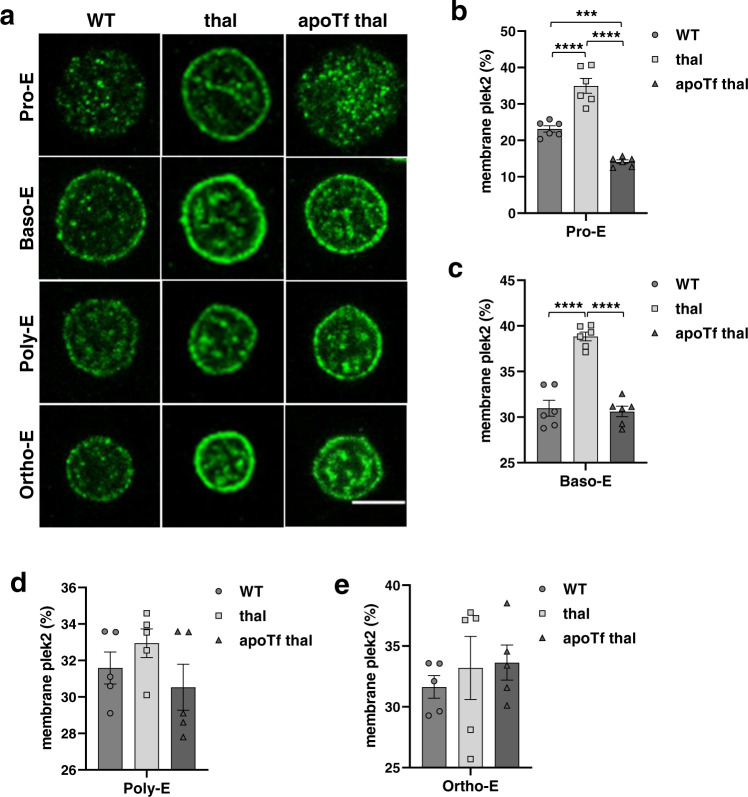
Early pleckstrin-2 activation and membrane localization during β-thalassemic erythroid precursor differentiation. **a** Confocal immunofluorescence microscopy of plek2 (green) localization in sorted (2–3 mice pooled per data point, *n* = 3 data points per category) WT, β-thalassemic, and apoTf-treated β-thalassemic erythroblasts demonstrate an increased percentage of membrane plek2 localization relative to total plek2 in β-thalassemic relative to: **b** WT Pro-E and **c** Baso-E from mice, normalized in apoTf-treated β-thalassemic Pro-E (**b**) and Baso-E (**c**) with no differences in: **d** Poly-E and **e** Ortho-E. The confocal images were representative optical sections of the cell from at least 50 cells in each condition in 2 different experiments. Scale bar 5 µm. All data are reported as mean ± s.e.m. and *p* < 0.05 was considered statistically significant (one-way ANOVA) (*p ≤ 0.05, **p ≤ 0.005, ***p ≤ 0.0005). WT = wild type; apoTf = apo-transferrin; plek2 = pleckstrin-2; Pro-E = pro-erythroblasts; Baso-E = basophilic erythroblasts; Poly-E = polychromatophilic erythroblasts; Ortho-E = orthochromatophilic erythroblasts.

We next set out to explore the mechanism of plek2 effect on apoptosis and enucleation in β-thalassemic erythroblasts. Plek2 is important for cytoskeletal integrity during erythropoiesis^24,26^. Plek2 membrane localization requires phosphoinositide 3-kinase (PI3K) downstream of Epo:EpoR signaling^27,36^, and PI3K-generated membrane-bound phospholipids recruit plek2 to the cell membrane where it is functionally active^36^. We hypothesize that in addition to increased plek2 protein expression, the high Epo level in β-thalassemia increases plek2 erythroblast membrane localization. Using immunofluorescence, we determine for the first time that in sorted WT bone marrow erythroblasts, plek2 membrane localization increases subsequent to Pro-Es (Supplementary Fig. 5a), but plek2 is already membrane associated in Pro-Es in β-thalassemic erythroblasts (Fig. 4a), decreasing after the Baso-E stage (Supplementary Fig. 5b). Quantification of the percentage of plek2 membrane localization demonstrates an earlier activation (Fig. 4b) and 2-fold increase of membrane plek2 in early erythropoiesis (Fig. 4b, c) in β-thalassemic mice relative to WT, which is normalized in apoTf-treated β-thalassemic erythroblasts (Fig. 4b, c). No differences in plek2 membrane localization are evident in later stages (Fig. 4d, e), reinforcing the importance of plek2 expression, membrane localization, and consequent activation at earlier stages in β-thalassemic erythroblasts.

### Earlier activation of pleckstrin-2 decreases apoptosis in early stage β-thalassemic erythroblasts

To further confirm that plek2 functions by an Epo-downstream mechanism to prevent apoptosis in conditions of expanded erythropoiesis, we phlebotomy-induced stress erythropoiesis, demonstrating an increased number of bone marrow erythroid precursors (Supplementary Fig. 6a) and increased erythroblast ROS (Supplementary Fig. 6b) without increased apoptosis (Supplementary Fig. 6c). Quantification of immunofluorescence in phlebotomized WT erythroblasts again revealed 2-fold increased membrane plek2 localization (Supplementary Fig. 6d) in erythroblasts, confirming the dependence of plek2 activation on Epo/EpoR signaling and association between increased ROS without increased apoptosis.

**Fig. 5:**
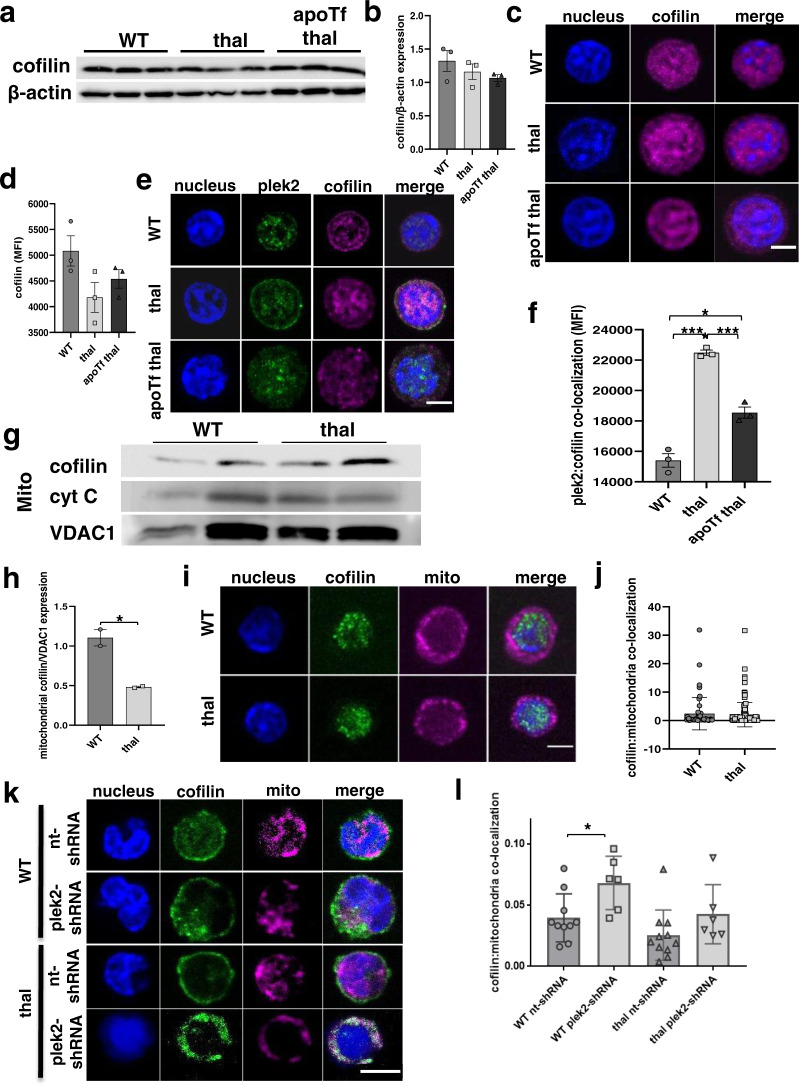
Pleckstrin-2 activation and cofilin binding prevents mitochondrial translocation of cofilin in β-thalassemic erythroblasts. **a** Western blot analysis of cofilin protein concentration in bone marrow erythroblasts from WT, β-thalassemic, and apoTf β-thalassemic mice (*n* = 3 mice/group; MW = cofilin 19 kDa, actin 42 kDa). **b** Statistical analysis of (**a**) demonstrates no differences in cofilin protein concentration between erythroblasts from WT, β-thalassemic, and apoTf β-thalassemic erythroblasts. **c** Confocal immunofluorescence microscopy of cofilin expression in sorted (2–3 mice pooled per data point, *n* = 3–4 data points per category) Pro-Es from WT, β-thalassemic, and apoTf-treated β-thalassemic mice. **d** Statistical analysis of cofilin MFI in (**c**) demonstrates no differences between Pro-Es from WT, β-thalassemic, and apoTf-treated β-thalassemic mice. **e** Confocal immunofluorescence microscopy of plek2 and cofilin co-localization in sorted (2–3 mice pooled per data point, *n* = 3–4 data points per category) Pro-E from WT, β-thalassemic, and apoTf-treated β-thalassemic mice. The confocal images are representative optical sections of at least 50 cells in each condition in 2 different experiments. Scale bar 5 µm. **f** MFI of plek2:cofilin co-localizations in Pro-Es from WT, β-thalassemic, and apoTf-treated β-thalassemic mice demonstrates higher co-localization predominantly in β-thalassemic relative to WT, normalized after apoTf treatment. All data are reported as mean ± s.e.m. and *p* < 0.05 (one-way ANOVA) is considered statistically significant. **g** Western blot analysis and **h** quantification of mitochondrial cofilin expression demonstrates no differences in cofilin translocation to the mitochondria in bone marrow erythroblasts from β-thalassemic relative to WT mice. (MW = cofilin 19 kDa, cytochrome *c* 14 kDa, VDAC1 35 kDa). **i** Confocal immunofluorescence microscopy of cofilin (green) and mitochondria (magenta) co-localization in Pro-E from WT and β-thalassemic mice. Scale bar 30 µm. The confocal images are representative of at least 20 cells analyzed in each condition. **j** Statistical analysis of (**i**) demonstrates no difference in cofilin/mitochondria co-localization in β-thalassemic erythroblasts compared to WT. **k** Confocal immunofluorescence microscopy of cofilin (green) and mitochondria (magenta) co-localization in fetal liver cells from WT and β-thalassemic mice transfected with either nt-shRNA or plek2-shRNA. Scale bar 50 µm. The confocal images are representative of at least 20 cells analyzed in each condition. **l** Statistical analysis of (**k**) demonstrates borderline decreased cofilin/mitochondria co-localization in nt-shRNA β-thalassemic erythroblasts compared to nt-shRNA WT (*p* = 0.06) as well as increased cofilin/mitochondria co-localization in both WT and borderline increased cofilin/mitochondria co-localization β-thalassemic (*p* = 0.07) erythroblasts transfected with plek2-shRNA compared to their respective controls (nt-shRNA). All data are reported as mean ± s.e.m. and *p* < 0.05 was considered statistically significant (Student’s unpaired *t*-test) (*p ≤ 0.05, **p ≤ 0.005, ***p ≤ 0.0005). WT = wild type; apoTf = apo-transferrin; plek2 = pleckstrin-2; Pro-E = pro-erythroblasts; Baso-E = basophilic erythroblasts; Poly-E = polychromatophilic erythroblasts; Ortho-E = orthochromatophilic erythroblasts; MFI = mean fluorescent index.

We next hypothesized that an increase in membrane-associated and activated plek2 protects β-thalassemic erythroblasts from obligatory high ROS in early stage erythroblasts (Fig. 1a) and accounts for the lack of increased apoptosis (Fig. 2g) in β-thalassemic relative to WT erythroblasts (Fig. 2h)^13^. Recently published evidence suggested that plek2 binds cofilin to prevent cofilin translocation to the mitochondria to prevent erythroblast apoptosis in the setting of increased ROS^24^. We thus explored the effect of increased plek2 expression, membrane localization, and activation on cofilin expression and localization in β-thalassemic mouse erythroblasts. Our results demonstrate that cofilin protein concentration does not differ between WT, β-thalassemic, and apoTf-treated β-thalassemic mice in bone marrow erythroblasts as a whole (Fig. 5a, b; and full blot Supplementary Fig. 10) or more specifically in Pro-E (Fig. 5c, d), with a decrease in expression after apoTf treatment only in later stage erythroblasts (Supplementary Fig. 7a, b). However, plek2:cofilin co-localization is higher in β-thalassemic Pro-Es, partially restored in apoTf-treated β-thalassemic mice (Fig. 5e, f); no differences in plek2:cofilin co-localization is evident in late stages of terminal erythropoiesis (Supplementary Fig. 7c, d). These results support the rationale for the functional significance of increased membrane localization and consequent activated plek2 also in β-thalassemic erythroblasts, specifically in early stage erythroblasts when ROS levels are highest, and complement previously delineated mechanisms for plek2 function in normal erythropoiesis^24^.

To assess whether plek2 is involved in preventing ROS-induced apoptosis, we next analyze whether plek2 functions by binding cofilin also in β-thalassemic erythroblasts, by preventing cofilin translocation to the mitochondria in high-ROS states^24^. We demonstrate a decrease in mitochondrial cofilin in β-thalassemic relative to WT bone marrow erythroblasts as a whole (Fig. 5g, h; and full blot Supplementary Fig. 10), despite elevated ROS. We further evaluate the effect of plek2:cofilin interaction on cofilin mitochondrial translocation specifically in Pro-Es, in which ROS and plek2:cofilin co-localization are highest. We demonstrate that mitochondrial cofilin is not increased in β-thalassemic relative to WT Pro-Es (Fig. 5i, j). We also analyze mitochondrial cofilin in vitro in nt-shRNA and plek2-shRNA transduced WT and β-thalassemic FLCs. We demonstrate that mitochondrial cofilin is borderline decreased in β-thalassemic relative to WT nt-shRNA transduced FLCs, increased in WT plek2-shRNA (vs. nt-shRNA) transduced FLCs, and borderline increased in β-thalassemic plek2-shRNA (vs. nt-shRNA) transduced FLCs (Fig. 5k, l). These findings confirm that mitochondrial cofilin in β-thalassemic erythroblasts does not increase despite increased ROS in vivo, is borderline decreased relative to WT erythroblasts in vitro, and that mitochondrial cofilin is increased in plek2 knockdown experiments in early stage erythroblasts in vitro. Overall, these results demonstrate that plek2—via its known interaction with cofilin, preventing apoptosis in high-ROS states by blocking mitochondrial translocation of cofilin—serves a compensatory anti-apoptotic function in β-thalassemic erythroblasts.

### Pleckstrin-2 binds and activates RacGTPase to enhance erythroblast enucleation in β-thalassemic mice

**Fig. 6:**
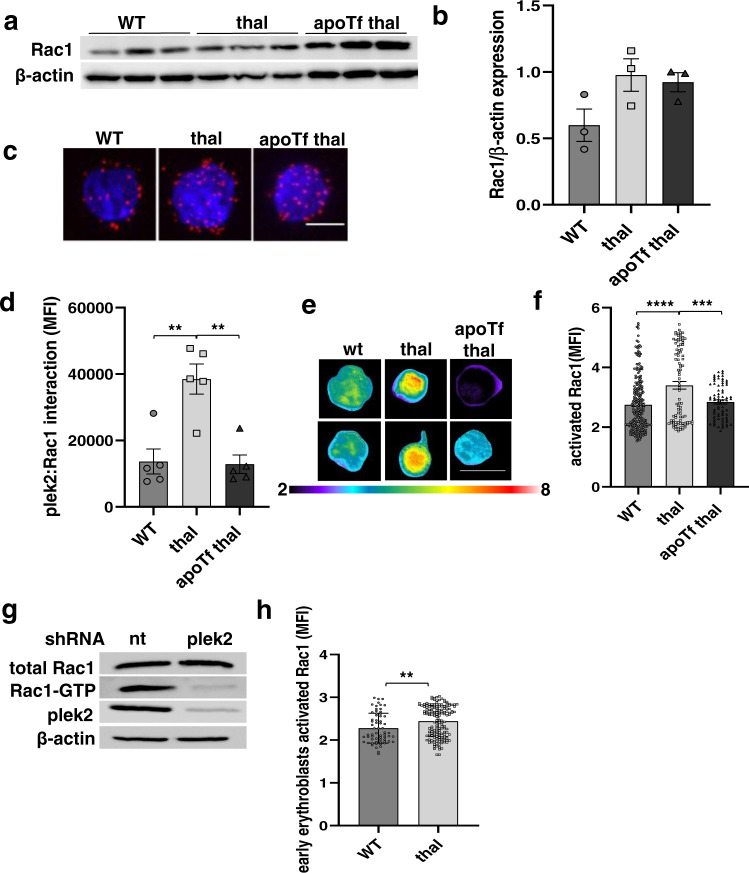
Pleckstrin-2 interacts with and results in RacGTPase activation in erythroid precursors. **a** Western blot for Rac1 expression in bone marrow erythroblasts from WT, β-thalassemic, and apoTf-treated β-thalassemic mice. **b** Statistical analysis of (**a**) reveals no difference in Rac1 expression between groups (*n* = 3 mice/group; MW = Rac1 21 kDa, actin 42 kDa). **c** Confocal immunofluorescence microscopy of Rac1 and plek2 interaction (red) using the proximity ligation assay in sorted bone marrow Pro-Es from (2–3 mice pooled per data point, *n* = 3 data points per category) WT, β-thalassemic, and apoTf-treated β-thalassemic mice. Scale bar 5 µm. **d** Statistical analysis of (**c**) quantifying Rac1 and plek2 interaction, demonstrating increased interaction in Pro-E from β-thalassemic relative to WT mice, normalized after apoTf treatment. **e** FRET of activated Rac1 in erythroblasts from WT, β-thalassemic, and apoTf-treated β-thalassemic mice. Scale bar 10 µm. The images are representative cells from at least 20 cells analyzed in each condition. **f** Statistical analysis of (**e**) reveals more activated Rac1 in β-thalassemic relative to WT erythroblasts, normalized after apoTf treatment. All data are reported as mean ± s.e.m. and *p* < 0.05 is considered statistically significant (one-way ANOVA). **g** Activated Rac1 pull-down from fetal liver cells from E13.5 WT embryos transfected with nt-shRNA and plek2-shRNA (MW = plek2 38 kDa, Rac1 21 kDa, actin 42 kDa). **h** Statistical analysis of activated Rac1 FRET in WT and β-thalassemic erythroblasts (*n* = 4 mice/group) cultured for 24 h (early erythroblasts) reveals more activated Rac1 in β-thalassemic relative to WT. All data are reported as mean ± s.e.m. and *p* < 0.05 is considered statistically significant (Student’s unpaired *t*-test). All data are reported as mean ± s.e.m. and *p* < 0.05 was considered statistically significant (two-way ANOVA) (*p ≤ 0.05, **p ≤ 0.005, ***p ≤ 0.0005). WT = wild type; thal = β-thalassemic; apoTf = apo-transferrin; plek2 = pleckstrin-2; MFI = mean florescence index; Pro-E = pro-erythroblasts; Baso-E = basophilic erythroblasts; Poly-E = polychromatophilic erythroblasts; Ortho-E = orthochromatophilic erythroblasts; FLC = fetal liver cell.

A role for plek2 in enucleation has also been postulated^24^, and other PH-domain-containing proteins have been shown to promote activated Rac formation, a central molecular switch in many signaling pathways^37^. Because β-thalassemic erythroblasts exhibit an enucleation defect^31^, we next evaluate mechanism by which plek2 impacts enucleation in β-thalassemic erythroblasts. Because RacGTPases are essential in erythroblast enucleation^9^, we hypothesize that increased β-thalassemic erythroblast plek2 expression, membrane localization, and consequence activation enhances Rac1 activation through increased plek2 and Rac1 interaction^36^. To address this hypothesis, we first explore Rac1 protein expression, demonstrating no changes in β-thalassemic relative to WT and apoTf-treated β-thalassemic bone marrow erythroblasts as a whole (Fig. 6a, b; and full blot Supplementary Fig. 10). We then evaluate plek2 and Rac1 interaction by performing the well-established proximity ligation assay (PLA)^38–40^ specifically in Pro-E. We demonstrate increased plek2:Rac1 interaction in β-thalassemic relative to WT Pro-E which is normalized after apoTf treatment (Fig. 6c, d), with no differences in later erythroblast stages (Supplementary Fig. 8a, b).

We next hypothesized that plek2:Rac1 interaction influences Rac1 activity in early stage erythroblasts. To explore this hypothesis, we quantified Rac1 activity by transfecting erythroblast from WT, β-thalassemic, and apoTf-treated β-thalassemic mice with a biosensor for activated Rac1^41^. This approach enables quantification of FRET signal, another well-established method for determining protein–protein interaction. We demonstrate that Rac1 activation is increased in β-thalassemic bone marrow erythroblasts as a whole compared to WT and normalized after apoTf-treatment (Fig. 6e, f). Taken together, these findings confirm a strong correlation between plek2:Rac1 interaction and Rac1 activation also in β-thalassemic erythroblasts.

Finally, to evaluate if the increased Rac1 activation is a direct consequence of increased plek2:Rac1 interaction, we performed a pull-down of activated Rac1 from WT FLC transfected with either nt-shRNA or plek2-shRNA, demonstrating decreased Rac1 activation in plek2-shRNA transfected WT FLC compared to the control (Fig. 6g; and full blot Supplementary Fig. 10). To clarify the significance of Rac1 activation in early and late stages of terminal erythropoiesis in vivo, FLCs cultured for 24 h (early erythroblasts) and 48 h (late erythroblasts) demonstrate increased activated Rac1 only in early (Fig. 6h) but not later stage (Supplementary Fig. 8c) β-thalassemic erythroblasts, again underlying the importance of changes in early stage of erythropoiesis. Lastly, using a specific Rac1 inhibitor, NSC23766^7,35,42^, we evaluate the effect of Rac1 inhibition in both WT and β-thalassemic FLCs in early stage erythroblasts. The effect of NSC23766 on enucleation is dose dependent but does not disproportionately impact β-thalassemic relative to WT erythroblasts (Supplementary Fig. 9), confirming that plek2 enhances Rac1 activation and prevents worsening of Rac1-mediated enucleation in β-thalassemic erythroblasts.

## Discussion

In the current work, we utilize the reversal of ineffective erythropoiesis in apoTf-treated β-thalassemic mice as a tool to evaluate the regulatory nodes downstream of Epo to gain a mechanistic understanding of dysfunctional erythropoiesis and its compensation in β-thalassemia. Taken together, our findings demonstrate that, while the loss of plek2 leads to a mild phenotype in a WT background^25^, plek2 loss in β-thalassemic mice results in an embryonic lethal phenotype with worsening ineffective erythropoiesis and more apoptosis in vivo as well as decreased enucleation in vitro, confirming an important compensatory function of plek2 in β-thalassemic erythropoiesis.

Currently, understanding of what causes ineffective erythropoiesis is limited to a disease-causing impairment of erythroid differentiation^28^ and erythroblast apoptosis, possibly resulting from increased ROS and/or hemichrome deposition in β-thalassemia, leading to anemia. As a consequence, anemia results in increased serum Epo, leading to an anti-apoptosis effect and expanded erythropoiesis^43,44^ without recovery from anemia. Recent evidence indicates that Epo and iron are required for ROS generation in erythroblasts, and that ROS are necessary for terminal erythropoiesis^29^. However, because unchecked ROS accumulation results in shortened RBC survival and anemia^45–49^, ROS generation is both critical and potentially toxic, requiring significant coordination during erythropoiesis with particular importance for mitigating increased ROS in conditions of ineffective erythropoiesis as in β-thalassemia.

**Fig. 7:**
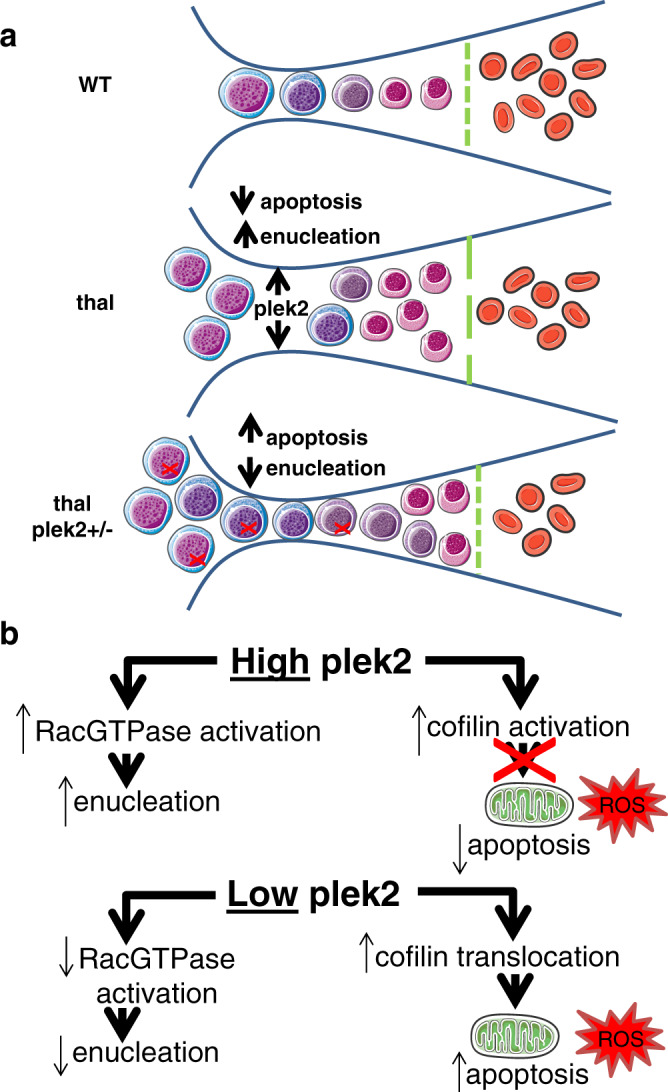
Mechanism by which pleckstrin-2 supports proliferation, decreases apoptosis, and enhances enucleation in ineffective erythropoiesis. **a** The normal balance of differentiation and proliferation enables effective erythropoiesis in WT mouse bone marrow. In β-thalassemic bone marrows, ineffective erythropoiesis results from an imbalance, with relatively more proliferating erythroblasts that differentiate and enucleate to form mature RBCs. When ROS are increased in β-thalassemic erythroblasts, elevated Epo enables multiple downstream mechanisms. Plek2 serves as a mechanism downstream of Epo by which more β-thalassemic erythroblasts evade apoptosis and increase erythroblast enucleation (green line). In thal plek2+/− bone marrow, more erythroblasts undergo apoptosis (red X) and fewer enucleate (green line), resulting in worsening anemia compared with β-thalassemic mice. **b** When membrane-associated pleckstrin-2 is increased, it’s binding to cofilin and consequent prevention of cofilin translocation to the mitochondria results in decreased apoptosis despite elevation of ROS in β-thalassemic erythroblasts. In addition, interaction with and activation of Rac1 results in enhanced enucleation in high-pleckstrin-2 states. Thus, when membrane-associated pleckstrin-2 is decreased in the setting of increased erythroblast ROS, relatively more cofilin translocation to the mitochondria leads to apoptosis and decreased Rac1 activation results in relatively decreased enucleation, leading to worsening ineffective erythropoiesis in β-thalassemia. WT = wild type; thal = β-thalassemia; plek2 = pleckstrin-2; ROS = reactive oxygen species.

We and others previously demonstrate that erythroblast ROS are increased without increasing apoptosis in β-thalassemic mouse models^13,28^. Our current data confirm that ROS levels are highest in early stage erythroblasts, decrease during progressive stages of normal erythroid differentiation^29,50^, and decrease only in Pro-Es from apoTf-treated β-thalassemic mice. These results extend previously demonstrated function of plek2 in erythropoiesis—by interacting with cofilin, preventing cofilin’s translocation to the mitochondria, and inhibiting the apoptosis pathway in response to increased ROS^23,24^—to an even greater degree in β-thalassemic erythroblasts, and indicate that increased ROS in late stage erythroblasts are insufficient to induce ineffective erythropoiesis in β-thalassemia. Thus, decreased plek2 enables apoptosis in response to increased ROS in β-thalassemic erythroblasts (Fig. 7a, b).

Furthermore, Epo also activates RacGTPase, known to play an essential role in both early and late stages of terminal erythropoiesis^51,52^. RacGTPase activation blockade in early stage erythroblasts and erythroid lineage-specific Rac1/2 knockout mice leads to decreased erythroblast differentiation and induces apoptosis^51,53^. Treatment of later stage erythroblasts with mutant Rac1 or Rac2 dramatically inhibits enucleation, confirming in vivo findings of enucleation blockade in Rac1/2/3 knockout mice^54^. Previous reports, using fluorescent microscopy and co-IP, demonstrate that plek2 and Rac1 are in close proximity in a manner unaffected by the activation state of Rac^55^. We demonstrate for the first time that increased plek2:Rac1 interaction correlates with enhanced Rac1 activation during erythroid differentiation in β-thalassemic mice. We also show that plek2 knockdown impairs enucleation also in β-thalassemic erythroblasts, likely via the known effect of decreased Rac1 activation on enucleation. These experiments can be further extended using Rac agonists in plek2 knockout erythroblasts to evaluate whether impaired enucleation is rescued.

In conclusion, our findings provide compelling mechanistic evidence for a critical role of plek2 in preventing apoptosis and enhancing enucleation during β-thalassemic erythropoiesis and as a useful marker of ineffective erythropoiesis. In addition, these findings implicate plek2 as a viable target downstream of Epo signaling for development of therapeutics in multiple diseases of dysregulated erythropoiesis.

## Methods

### Mice

C57BL6 (WT) and β-thalassemic (*Hbb*^*th1/th1*^ and *Hbb*^*th3/+*^ (thal))^56,57^ mice on a mixed background used for in vivo and FLC-derived erythroblasts were originally purchased from The Jackson Laboratories. Plek2 heterozygous mice (plek2+/−) were generated in the laboratory of Dr. Charles Abrams (UPenn, Philadelphia, PA)^25^. All experiments were conducted using 3–4 month old gender-matched mice. Progeny of plek2+/− mice crossed with th3/+ mice were generated to characterize double heterozygote mice (thal plek2+/−). Mice were treated with 10 mg of human apoTf (CSL Behring, Kankakee, IL) via intraperitoneal injection for 20 days^58^. Phlebotomy was performed via retro-orbital puncture with an average total of 0.4 mL of blood removed over 2 days; mice were sacrificed 24 h after the second phlebotomy. All mice were bred, housed, and sacrificed in the animal facility under AAALAC guidelines; all studies were approved by the Institutional Animal Care and Use Committee. To enable sufficient number of cells, pooling of 2–3 mice per data point was required in all sorting experiments, including RNA sequencing, immunofluorescence, PLA, FRET, and subcellular fractionation analyses (see below).

### Flow cytometry

Bone marrow cells were incubated with anti-CD45 magnetic beads (Mylteni, 130-052-301) and incubated with anti-mouse TER119-phycoerythrin Cy7 (PE-Cy7) (BioLegend, 116222), CD44-allophycocyanin (APC) (Tonbo, Biosciences, 20-0441-U100), and CD71-PE (BD Pharmigen, 553267)^2,59^. Erythroblast ROS and apoptosis quantification were performed using immunostaining for ROS and activated caspase 3 (Invitrogen, C6827 and C10423, respectively). Samples were analyzed using either FACSCanto I or LSRFortessa flow cytometer (BD Biosciences). Cell sorting was performed on a CSML4 Cell Sorter (BD Biosciences) using DIVA Software.

### Quantitative real-time PCR

RNA was purified from bone marrow erythroblasts using PureLink RNA Mini Kit (Ambion, Life Technology, 12183020) and analyzed with SuperScript III Platinum SYBR Green One-Step quantitative real-time polymerase chain reaction kit (Invitrogen, 11736059) according to the manufacturer’s protocols. Primers used for the PCR are: plek2-forward AAAGACCTTTCTGGGCTCCT, plek2-reverse CCCTACTGGCCTGAGAAAGT; FasL-forward ACCGGTGGTATTTTTCATGG, FasL-reverse TTTAAGGCTTTGGTTGGTGA; Fam132b-forward ATGGGGCTGGAGAACAGC, Fam132b-reverse TGGCATTGTCCAAGAAGACA. Cycle threshold (Ct) values were calculated by the CFX Manager^TM^ Software. Relative gene expression levels were expressed as the difference in Ct values (ΔCt) of the target gene and the housekeeping gene GAPDH as described^60^ in each sample and normalized to GAPDH expression. All PCRs were performed in triplicate.

### RNA sequencing

Total RNA was extracted from freshly sorted bone marrow Pro-E and stored at −80 °C until processing. The library preparation was performed using polyA^+^-enriched RNA according to the manufacturer’s instructions (Illumina) and then sequenced on a Solexa sequencing cell. Reads were aligned using STAR against mm10 with –outFilterMultimapNmax 1 option. Differential expression analysis and FPKM (fragments for kilobase millions) levels were calculated using Homer and EdgeR using all 3 replicates for each experiment, counting reads within exons on both strands at the gene level. AnalyzeRepeats.pl rna mm10 -strand both -count exons –condenseGenes. Average FPKM values were calculated for each category using all 3 replicates for each group of sorted Pro-E.

### Western blot

Bone marrow and FLC-derived CD45^−^ erythroblasts were lysed with protease inhibitors (Protease Inhibitor Cocktail Tablets, Roche), quantified, separated by sodium dodecyl sulfate-polyacrylamide gel electrophoresis (SDS-PAGE) using 10–12% polyacrylamide gels, and transferred to nitrocellulose membrane (Bio-Rad). Membranes were blocked for 1 h in TBST (10 mM Tris-HCl, pH 8.0; 150 mM NaCl; 0.05% Tween 20) containing 5% skim milk or 5% BSA, followed by overnight incubation with commercially available primary antibodies (anti-plek2 1:1000 (Proteintech, 11685-1-AP); anti-cofilin 1:1000 (Santa Cruz, sc-376476); anti-Rac1 1:1000 (Cell Signaling Technology, 2465); anti-VDAC1 1:1000 (Millipore, MABN504); anti-cytochrome *c* 1:1000 (Abcam, ab90529); and anti-GAPDH 1:4000 (Invitrogen)). Blots were washed and incubated for 1 h at room temperature (RT) with the secondary antibody (horseradish peroxidase (HRP) conjugated (Thermo Scientific)). Immunoreactive bands were visualized by the enhanced chemiluminescence (ECL) method (Amersham Bioscience) according to standard procedures. For mitochondria fractionation and activated Rac1 pull-down (Thermo Scientific, 89,874 and 16,118, respectively), samples were processed according to the manufacturer’s instructions.

### Cell culture

FLCs were isolated from genotyped E13.5 embryos, dissociated and filtered. Total TER119-negative FLCs were isolated using biotin anti-mouse TER-119 antibody (1:100), infected with retroviruses (plek2-shRNA) and nt-shRNA (non-targeting control-shRNA), and seeded (at a cell density of 3 × 10^5^ cells/mL) in fibronectin-coated wells (BD Discovery Labware, Bedford, MA). To evaluate the effects on early stages of erythropoiesis, purified cells were cultured for 12 h in Epo-free media with stem cell factor (SCF), interleukin-6, and FTL3 ligand (SCF media) to maintain their progenitor status, maintain cell survival, and allow the expression of the infected shRNA. The cells were then transferred to Epo-containing media (Iscove’s modified Dulbecco’s medium (IMDM) containing 15% FBS, 1% detoxified BSA (Sigma-Aldrich), 200 μg/mL holo-transferrin (Sigma), 10 μg/mL recombinant human insulin (Sigma-Aldrich), 2 mM L-glutamine, 10.4 M mercaptoethanol, and 2 U/mL recombinant human Epo (Amgen)), and continually cultured for 2 days. To study late stage terminal erythropoiesis, cells were immediately cultured in Epo media after infection. Samples were collected at 24 and 48 h after resuspension in Epo-containing media for proliferation and enucleation analysis by flow cytometry, and for Rac pull-down. To specifically inhibit Rac1 and ROS in early stage of erythropoiesis, infected cells after expansion were treated with different concentrations of NSC23766 (Sigma, SML0952)^7,24,35,42^ or ROS scavenger N-acetyl-cysteine (NAC, Sigma, A7250).

### Immunofluorescence

A total of 2–3 × 10^5^ bone marrow cells or FLCs were seeded on glass coverslips coated with poly-L-lysine (Corning BioCoat^TM^ 12 mm diameter) in 24-well plates, fixed with 4% paraformaldehyde in PBS, pH 7.2 at RT for 20 min, and permeabilized with ice-cold 0.1% Triton X-100 in PBS for 10 min.

For mitochondrial staining, cells were resuspended in pre-warmed staining solution containing the MitoTracker probe (Invitrogen, M22426) before permeabilization/fixation. Cells were then stained with rabbit anti-plek2 (1:100 (Proteintech)), mouse anti-cofilin (1:250 (Santa Cruz)) antibodies overnight at 4 °C. After washing in PBS, Alexa Fluor^®^488 goat anti-rabbit IgG (1:500, Invitrogen), Alexa Fluor^®^647 donkey-anti-mouse IgG (1:500 (Jackson Immunoresearch)) were added for 1 h at RT. Nuclei were identified using DAPI-Antifade (Molecular Probes). For analyses, for each high-power field, regions of interest (ROI) were defined by creating binary image masks through automatic thresholding of DAPI and plek2 positive staining. Noise was filtered out by applying a median filter (3 × 3 pixel radius). A cytoplasmic ROI was created by subtracting the DAPI staining mask (nuclear ROI) from the plek2 mask, and each of these ROI masks were applied to the original plek2 staining images to separate nuclear and cytoplasmic staining within each high-power field.

Data were exported from ImageJ and used to generate histograms into Microsoft Excel software. Analysis for plek2 nuclear:cytoplasmic localization during erythroid differentiation was performed with nuclear:cytoplasmic ratio as a relative measure of plek2 nuclear localization^61^. Nuclear and cytoplasmic histogram data were first normalized for the total number of cells/nuclei included in the analysis and then comparison was made of the average of staining intensities. For cofilin expression analysis, each high field was quantified for the mean fluorescence intensity and normalized for the total number of cells/nuclei by ImageJ software.

For mitochondrial cofilin co-localization, for each high-power field, ROI were defined by creating binary image masks through thresholding. Each of these ROI masks were then compared, by the image calculator, and subjected to a 3D object counter to obtain the overlap volumes (volumes shared by cofilin and mitochondrial positive staining) by using ImageJ software.

Microscopy was performed on a TCS SP5 confocal microscope (Leica Microsystems Heidelberg, Manheim, Germany) equipped with an Apo CS Lambda Blue 63 × 1.40 NA oil objective. Images (1024 × 1024) were acquired at 700 Hz in frame sequential mode, 2.7 zoom. All microscope settings were identical to enable direct comparison between groups. All cells were analyzed at the 3 most central Z stacks per cell.

### Proximity ligation assay

Cells were prepared as per immunofluorescence as above. Two primary antibodies selected from two different host species (rabbit anti-plek2, 1:100 (Proteintech); mouse anti-Rac1, 1:250 (BD Bioscience, 610650)) are used together with the generic Duolink species-specific secondary antibodies containing unique DNA strands that template the hybridization of added oligonucleotides. When in close proximity (<40 nm), the oligonucleotides are ligated by a ligase to form a circular template. This template, still anchored to the antibody, is subsequently amplified and detected using complementary labeled oligonucleotide probes^62–64^. The resulting distinct spots were derived from single-molecule protein interaction events.

Quantifications of PLA mean fluorescence intensity per cell was performed by using ImageJ software. PLA was performed using Duolink^®^ In Situ Red Starter Kit Mouse/Rabbit (Sigma, DUO92101-1KT), according to the manufacturer’s instructions. Final resultant images were visualized using TCS SP5 confocal microscope (Leica Microsystems Heidelberg, Manheim, Germany) equipped with an Apo CS Lambda Blue 63 × 1.40 NA oil objective. Images (1024 × 1024) were acquired at 700 Hz in frame sequential mode, 2.7 zoom.

### Biosensor assay and FRET analysis

Mouse bone marrow erythroblasts were transfected with the biosensor expression construct, using jetPRIME DNA (0.75 μg of DNA for 2.25 μL of het prime for a 12-well plate) and siRNA transfection reagent (ThermoFisher). The cells were plated at 2 × 10^5^ cells/mL density on glass coverslips coated with poly-L-lysine (Corning BioCoat^TM^ 12 mm diameter) and cultured for 24 h to allow the expression of the biosensor. Cells were then fixed in 4% PFA and imaged using a Leica DMi8 widefield microscope (Leica Microsystems Heidelberg, Manheim, Germany) equipped with a Spectra X LED light source and a monochromatic camera DFC 9000 GT (Leica, 2048 × 2048 pixels, 16 bit). CFP and FRET images were acquired with a 63 × 1.40 NA OIL HC PL APO objective by exciting with a 440 nm LED line and using a 430/24 excitation filter, 455,515 double dichroic, and 470/24 or 535/30 emission filter for CFP or FRET detection, respectively^41^. For each image, background was subtracted in CFP and FRET channel. Then a binary mask was generated through intensity thresholding and was applied to the matched FRET channel and CFP image. Noise was filtered out with a median filter (radius = 10 pixel). Final FRET images were obtained by dividing FRET by CFP channel. A linear pseudo color lookup table was applied, and the ratio values were normalized to the lower scale value. Quantifications of FRET intensity was performed per cell with a standard measurement of a circular ROI set on 100 pixels^65^.

### Generation and transduction of retroviral particles

Retroviral constructs MSCV-IRES-GFP- plek2-shRNA and nt-shRNA were created using HEK293T cells transfected with plasmids and the retroviral packaging construct Pcl-Eco in a 2:1 ratio by TransIT-LT1 transfection reagent (Mirus, MIR2300) according to the manufacturer’s protocols^24^. Viral supernatants were collected after 48–72 h. Retroviral infection was performed by resuspending the purified Ter119-negative FLCs from both WT and th1/th1 mice in viral supernatants with 8 μg/mL polybrene (Sigma) and centrifuged at 1800 rpm for 1.5 h at 37 °C. After spin-infection, the viral supernatants were immediately removed and fresh media added.

### Active Rac1 pull-down and detection

The Active Rac1 Pull-Down and Detection Kit (Thermo Scientific) was used to according to the manufacturer’s instructions. Briefly, pellets from 1 × 10^7^ FLCs infected with both nt-shRNA and plek2-shRNA, as well as CD45^−^ bone marrow erythroblasts were lysed with Lysis/Binding/Wash buffer on ice for 5 min and the supernatants were transferred to a new tube. To ensure the pull-down procedures were working properly, lysates were incubated with 0.5 M EDTA pH 8 and 0.1 mM GTPyS (positive control) or 1 mM GDP (negative control), and the mixture incubated at 30 °C for 15 min with constant agitation. Supernatants were transferred into spin cups with glutathione resin and GST-human Pak1-PBD. The reaction mixtures were incubated at 4 °C for 1 h with gentle rocking, and the resins were then washed 3 times with 400 μL of Lysis/Binding/Wash buffer. Resins were incubated for 2 min with 50 μL of 2× reducing sample buffer and the eluted samples were then heated for 5 min at 95–100 °C and electrophoresed on a 12% acrylamide gel.

### Statistics and reproducibility

All data are reported as mean ± s.e.m. and *p* < 0.05 is considered statistically significant. Significance of differences is determined with either ANOVA or Student’s unpaired *t*-test as appropriate. All analyses and correlations were performed using GraphPad Prism 8. A minimum of *n* = 3 was used in all experiments and when feasible, experiments were repeated to ensure reproducibility.

### Reporting summary

Further information on research design is available in the Nature Research Reporting Summary linked to this article.

## Supplementary information

Supplementary information.

Supplementary Data 1.

Reporting summary.

Description of Additional Supplementary Files.

## Data Availability

The dataset that supports the findings of this study has been deposited in the GEO repository accession number GSE158839. Source data are available in Supplementary Data 1. All other data supporting the findings of this study are available within the main manuscript or its adjoining supplementary information files. Any additional clarification is available from the corresponding author on reasonable request.
