# Supplementary material for: Pleckstrin-2 is essential for erythropoiesis in β-thalassemic mice, reducing apoptosis and enhancing enucleation
Source: Commun Biol. 2021 May 3;4:517. doi: 10.1038/s42003-021-02046-9 (PMC8093212; doi:10.1038/s42003-021-02046-9)
Supplement: Supplementary file 1 — Supplementary information. [file 42003_2021_2046_MOESM1_ESM.pdf]

Suppl Figure 1

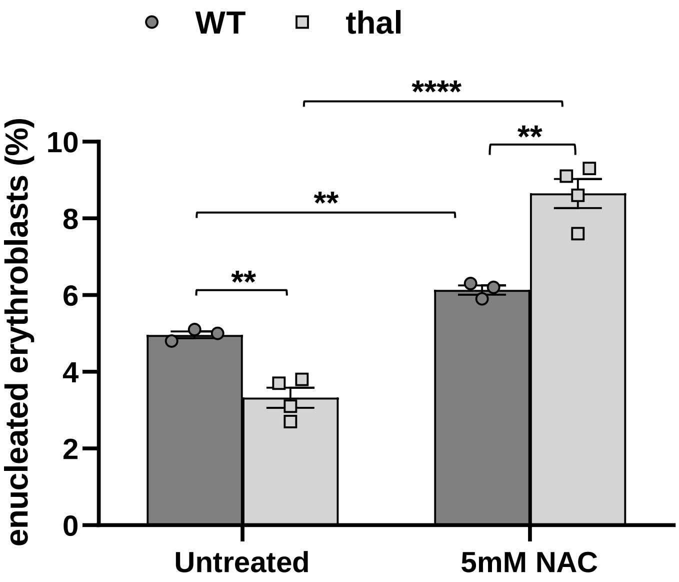

Suppl Figure 2

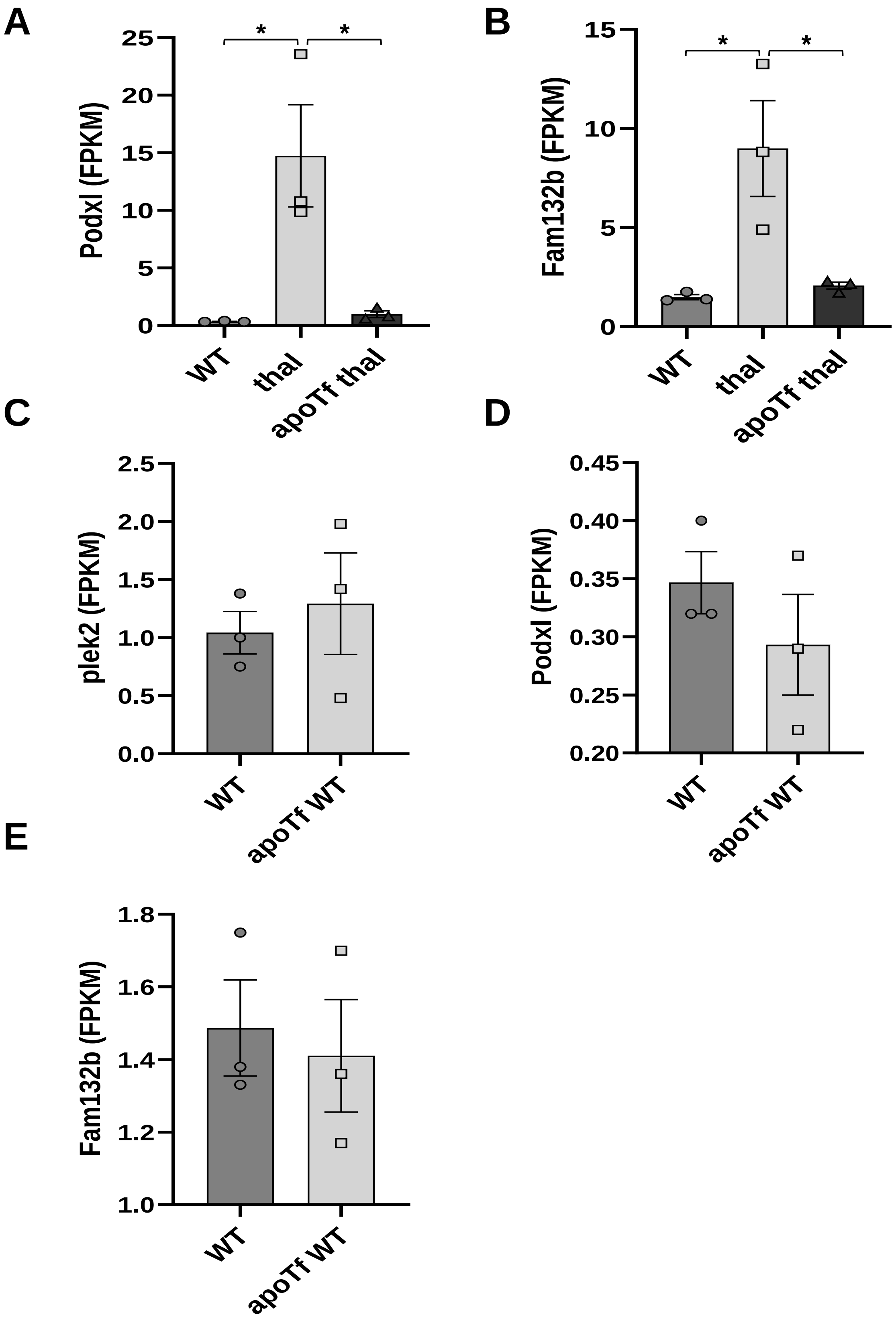

Suppl Figure 3

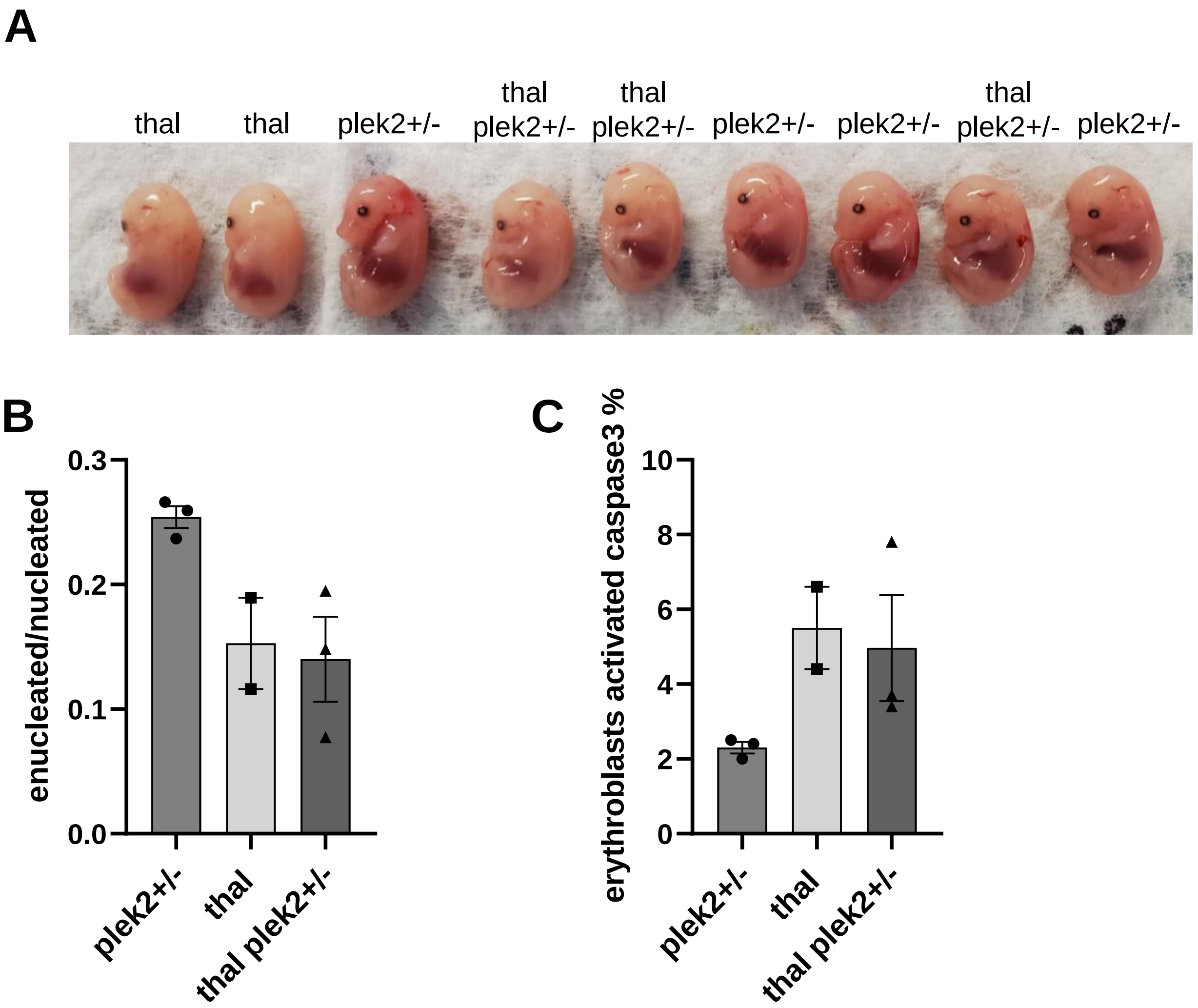

Suppl Figure 4

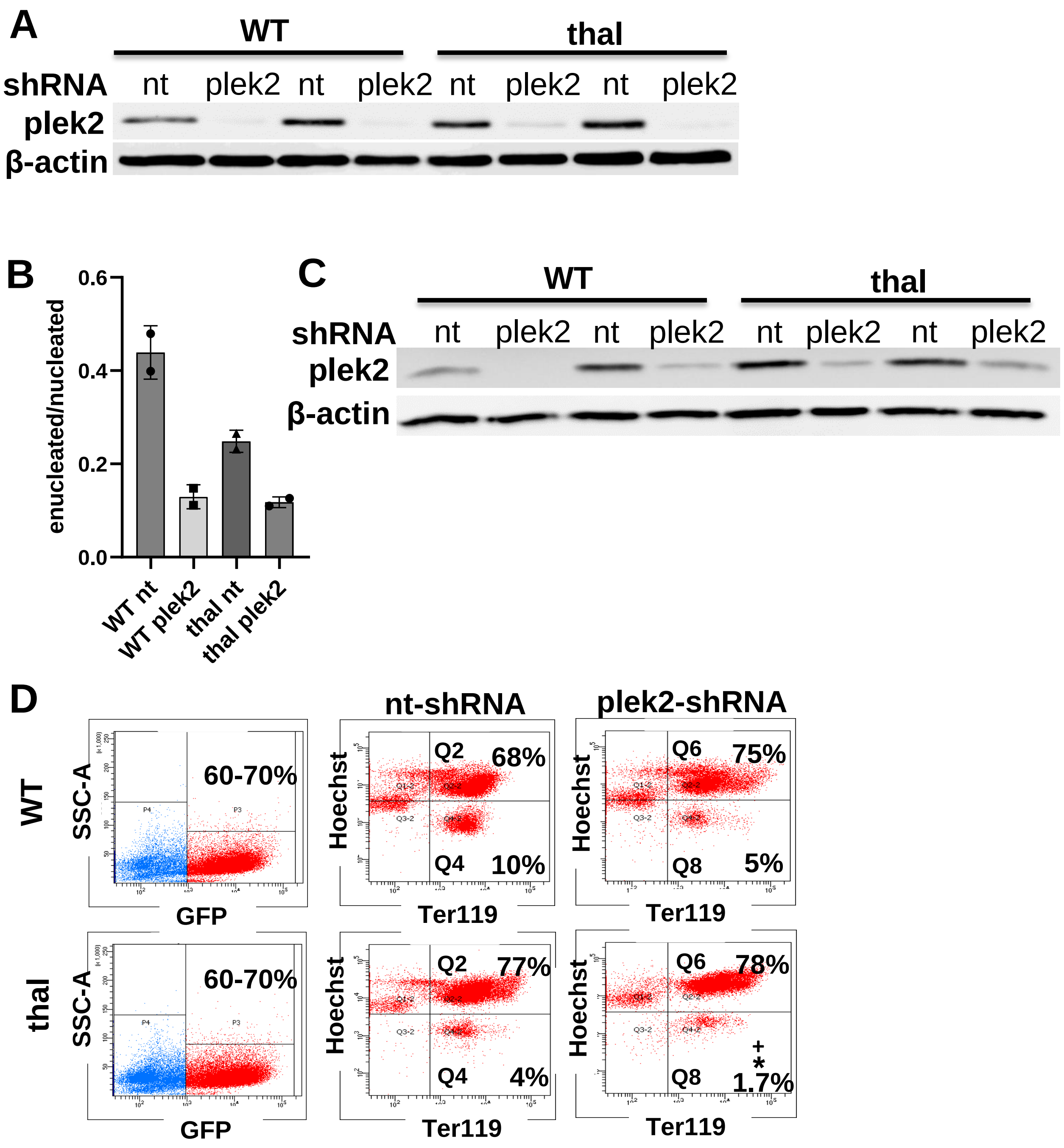

Suppl Figure 5

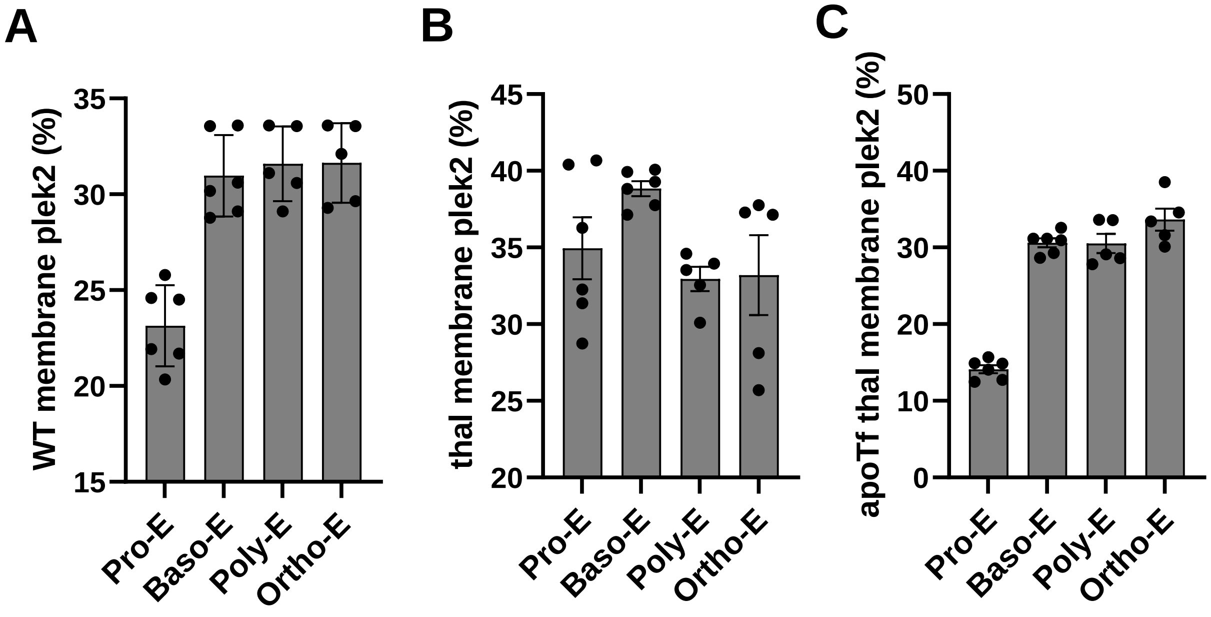

Suppl Figure 6

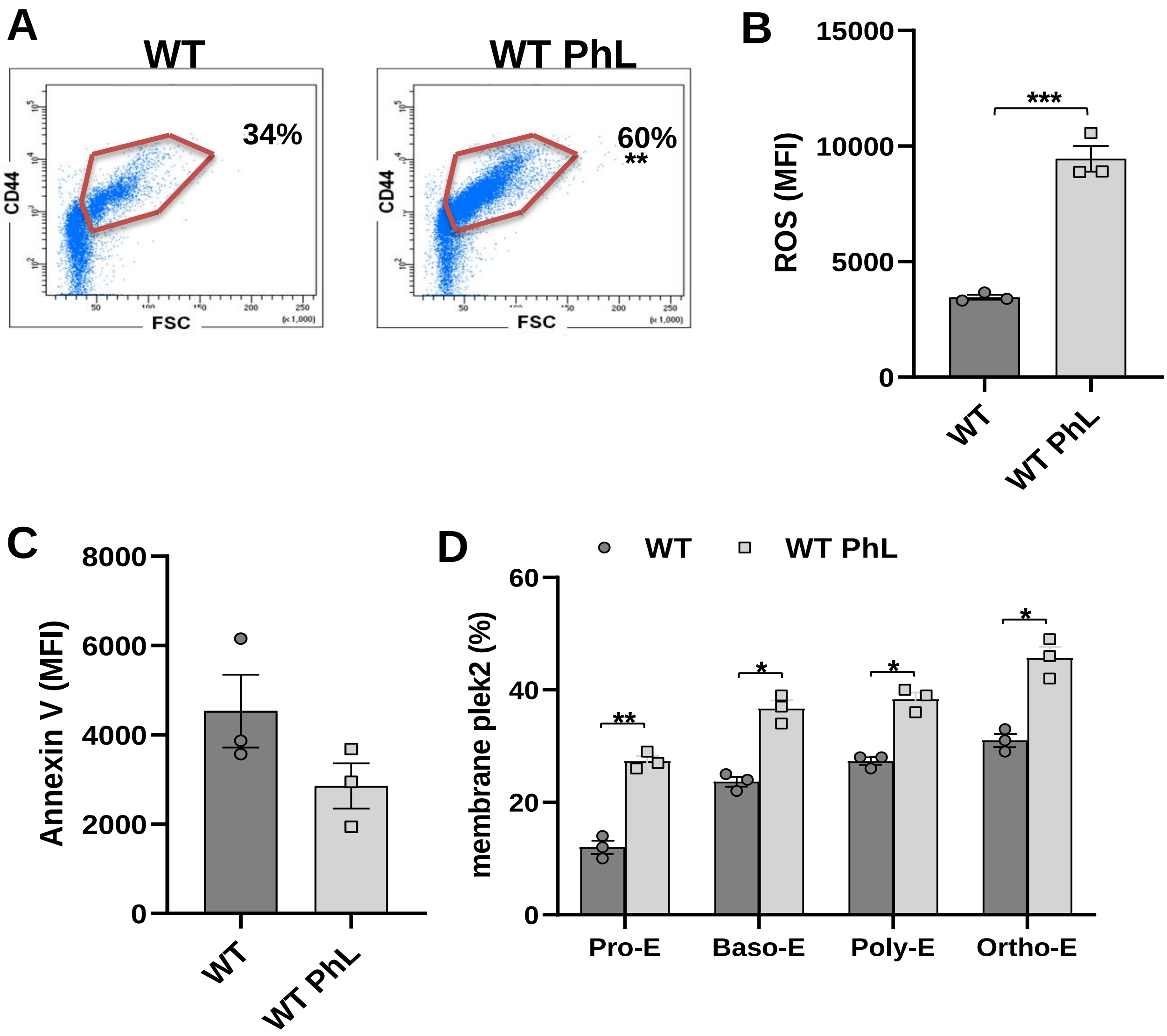

Suppl Figure 7

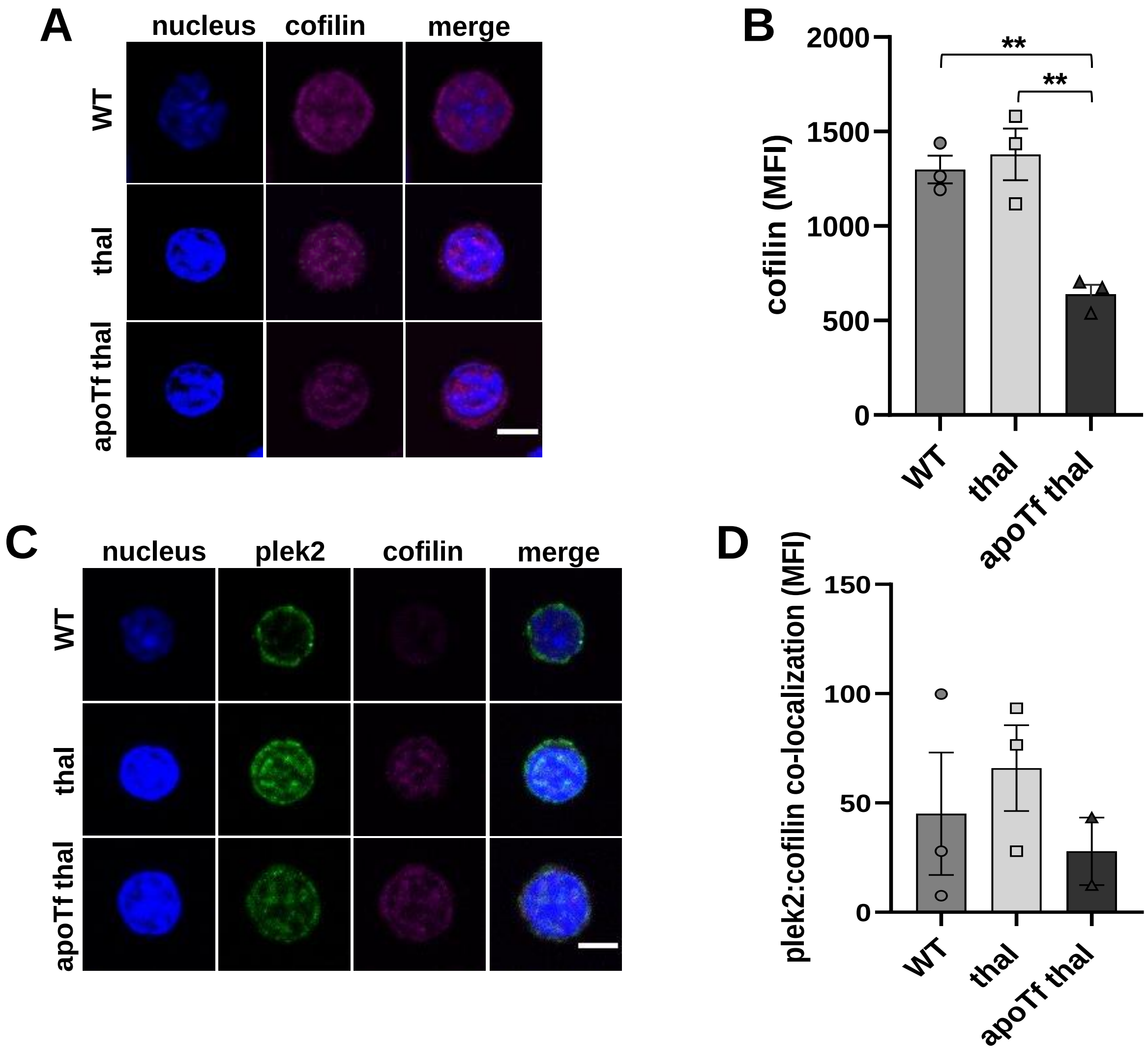

Suppl Figure 8

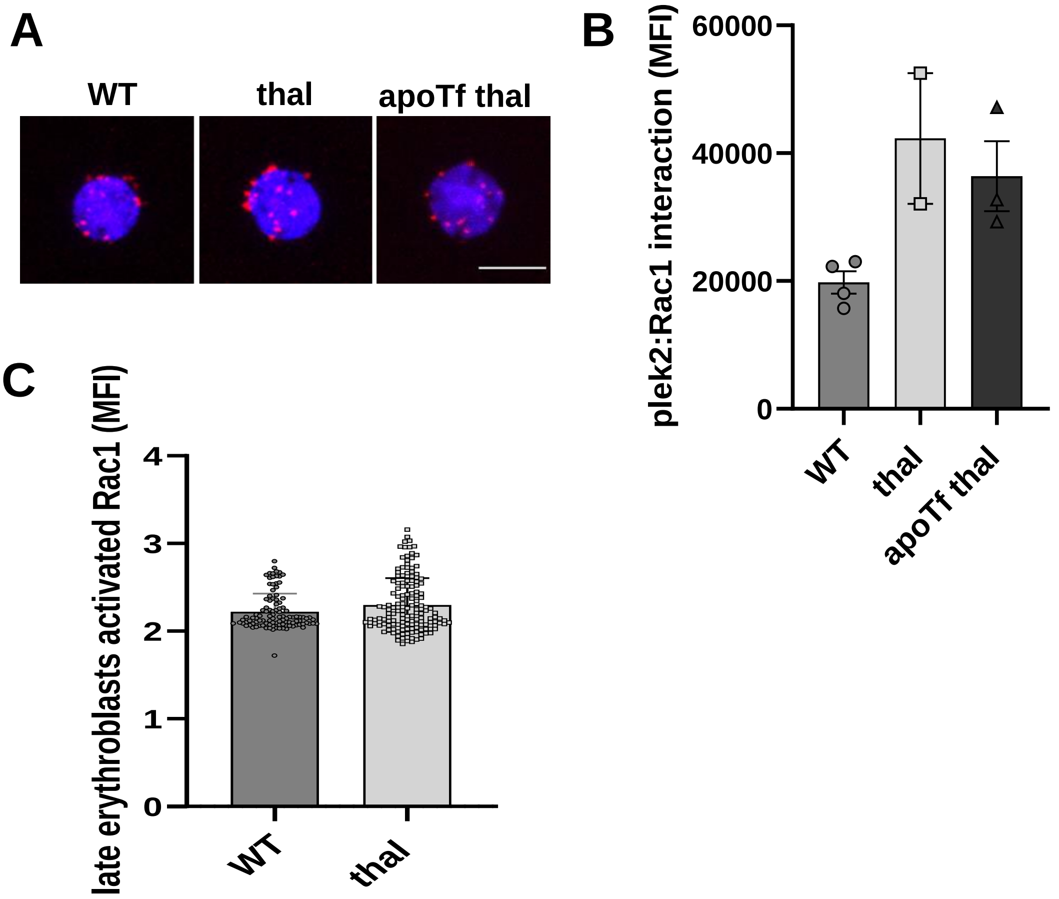

Suppl Figure 9

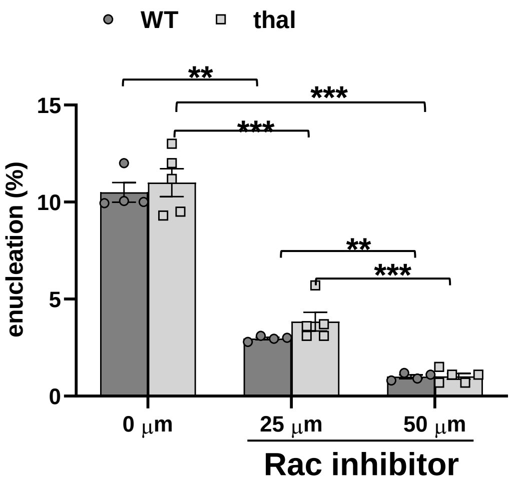

Suppl Figure 10

Figure 1f

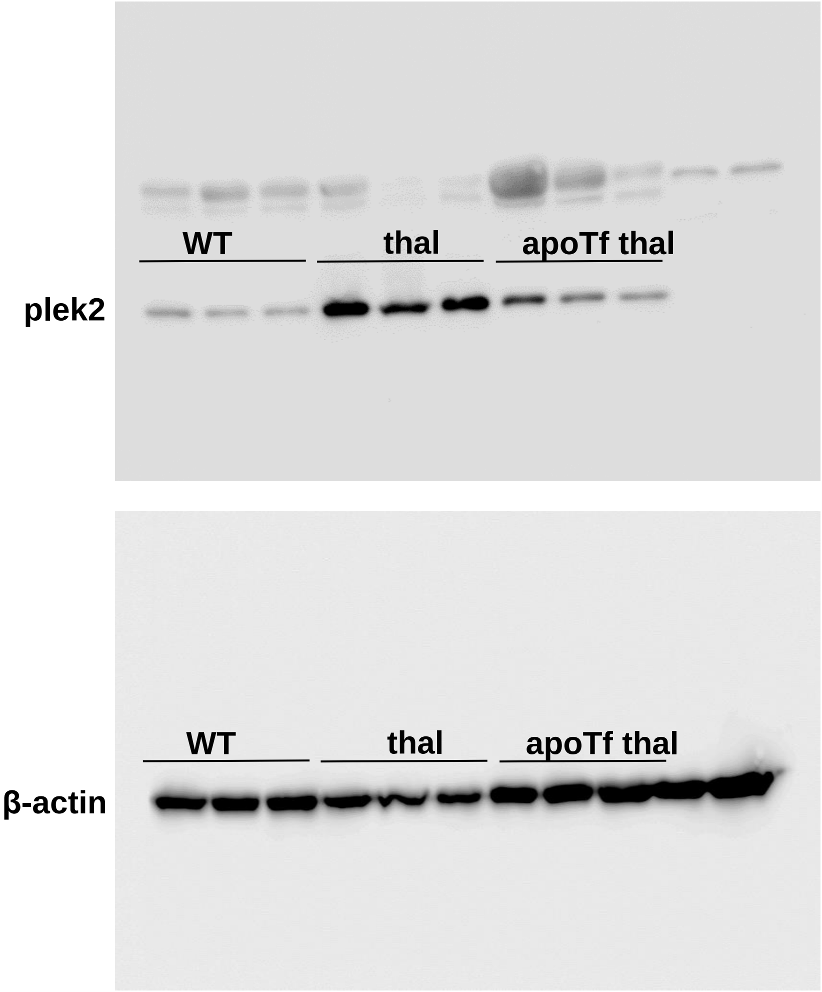

Figure 5a

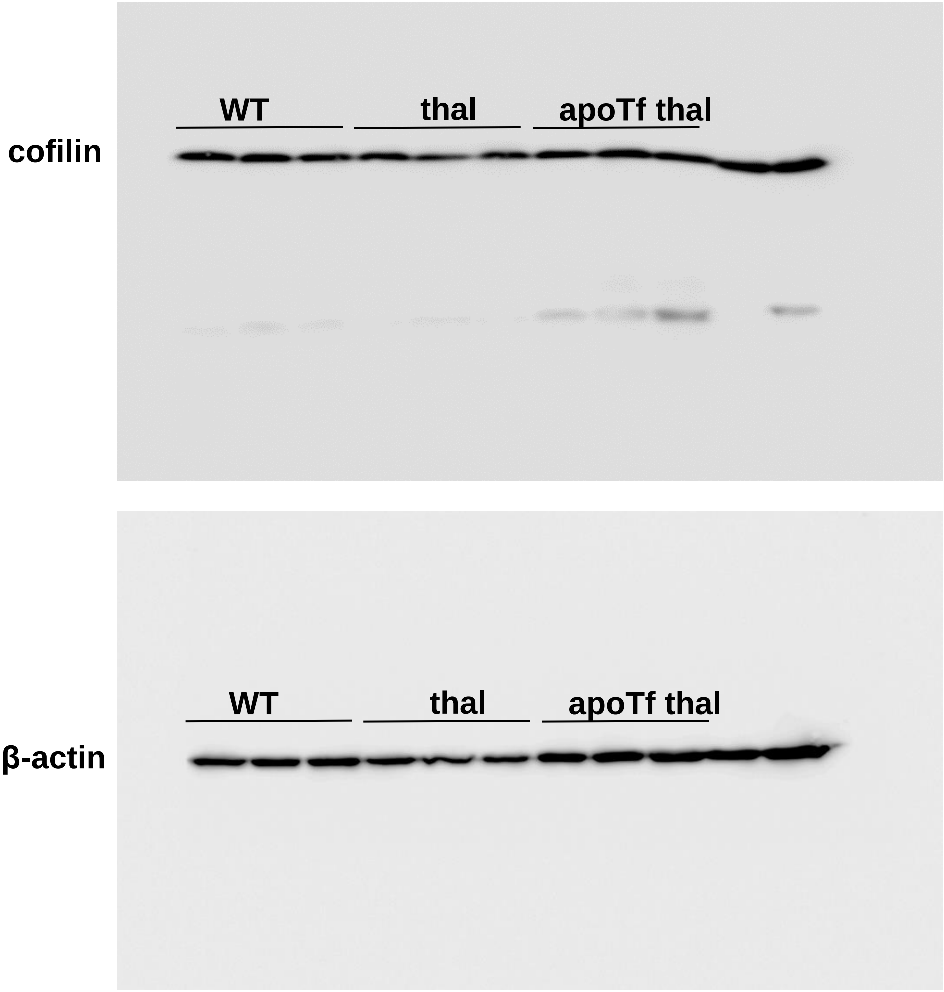

Figure 5g

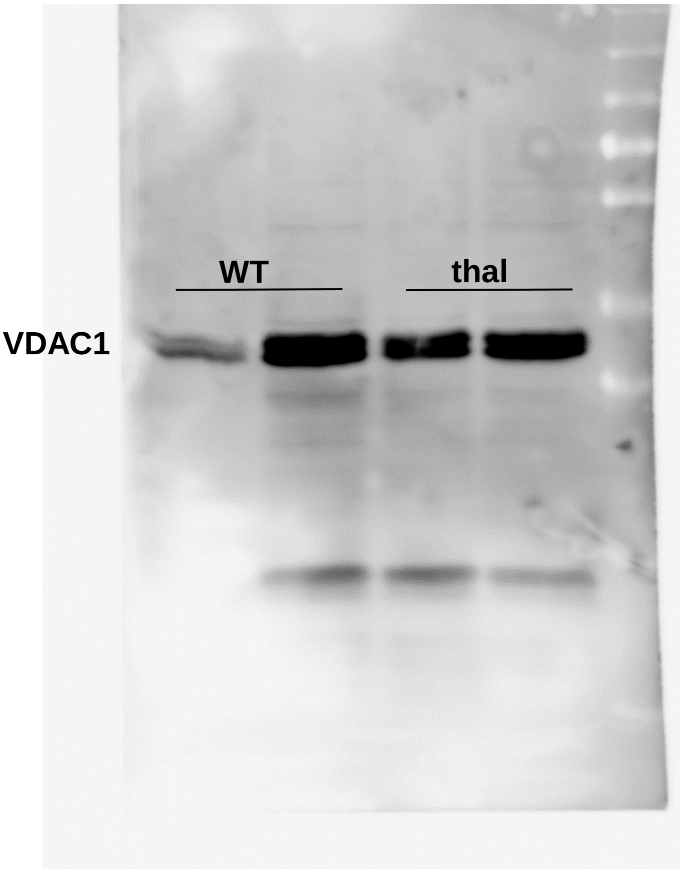

Figure 5g

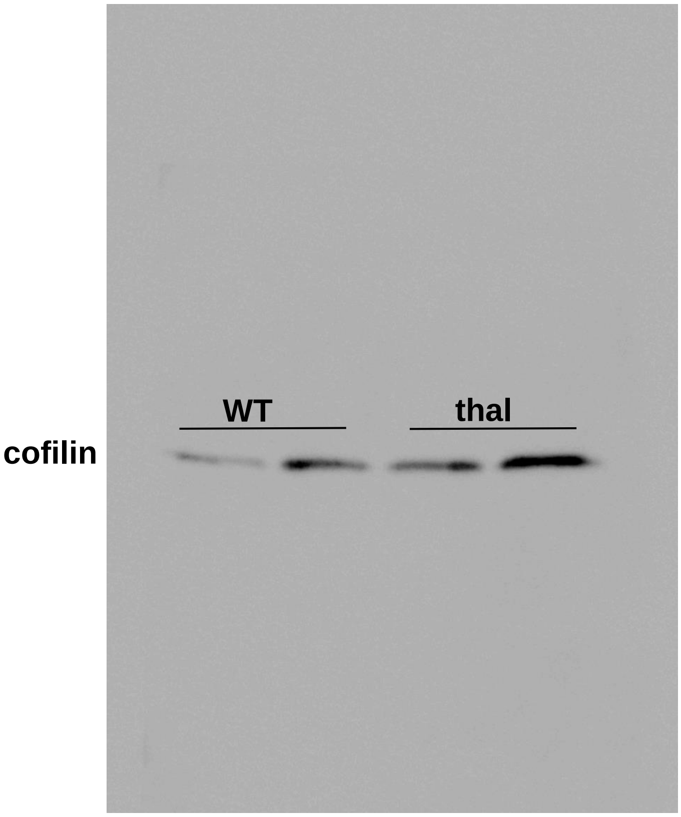

Figure 5g

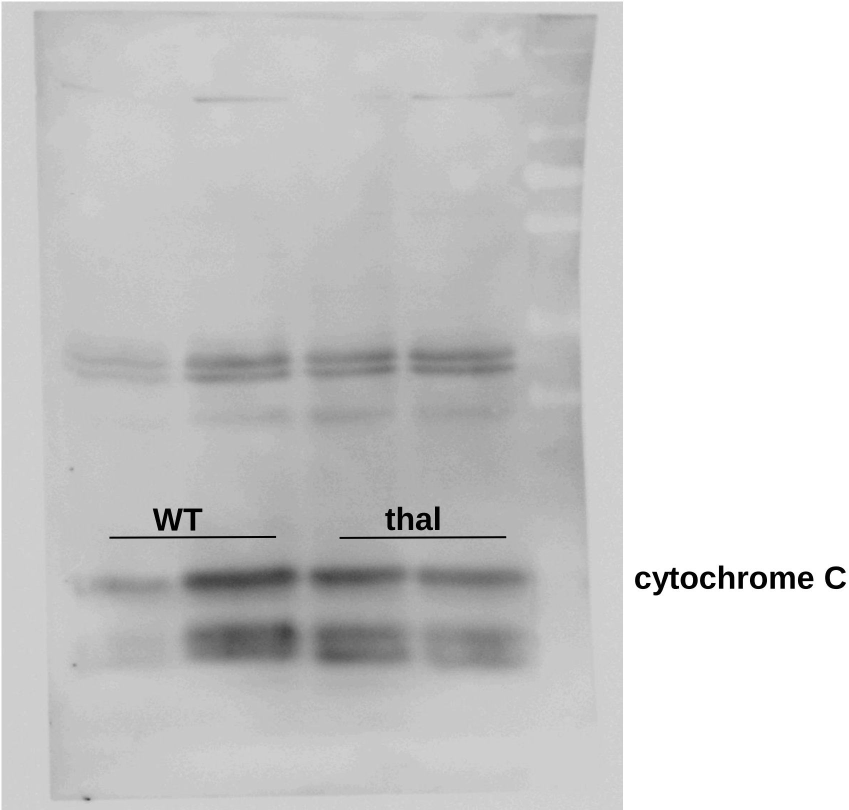

Figure 6a

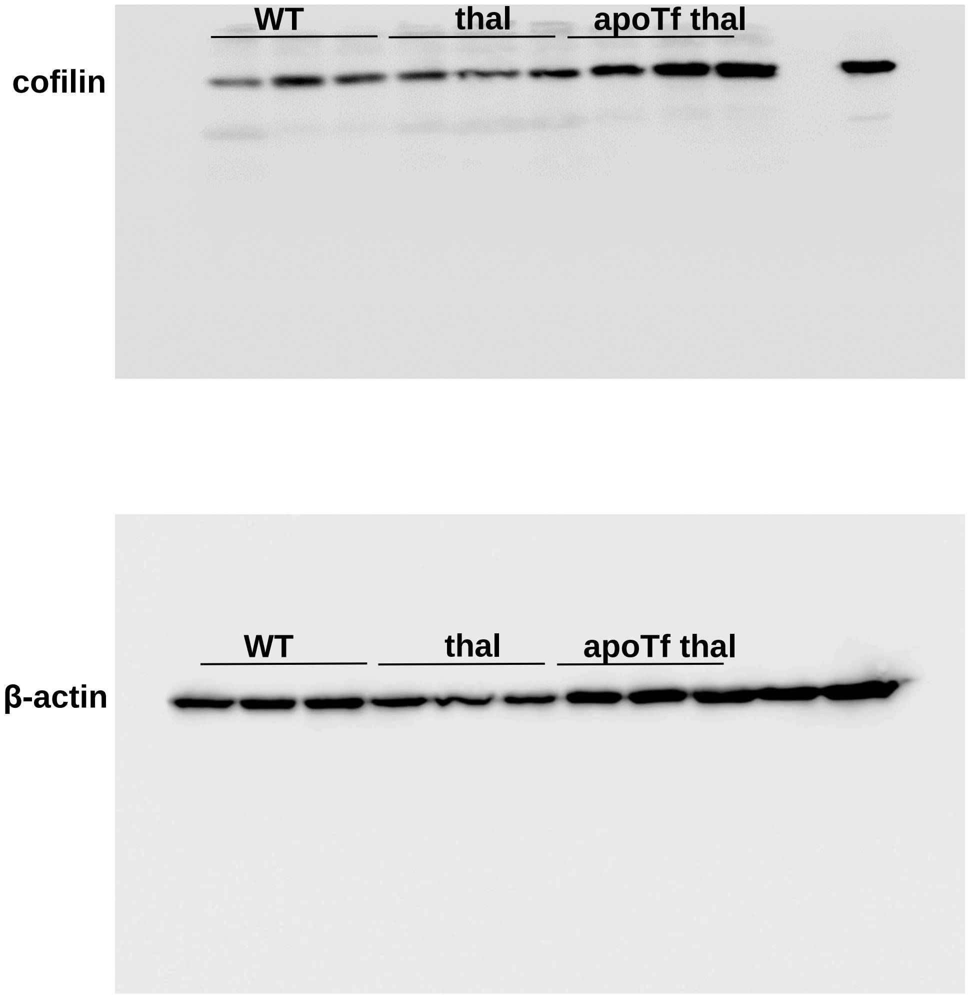

Figure 6g

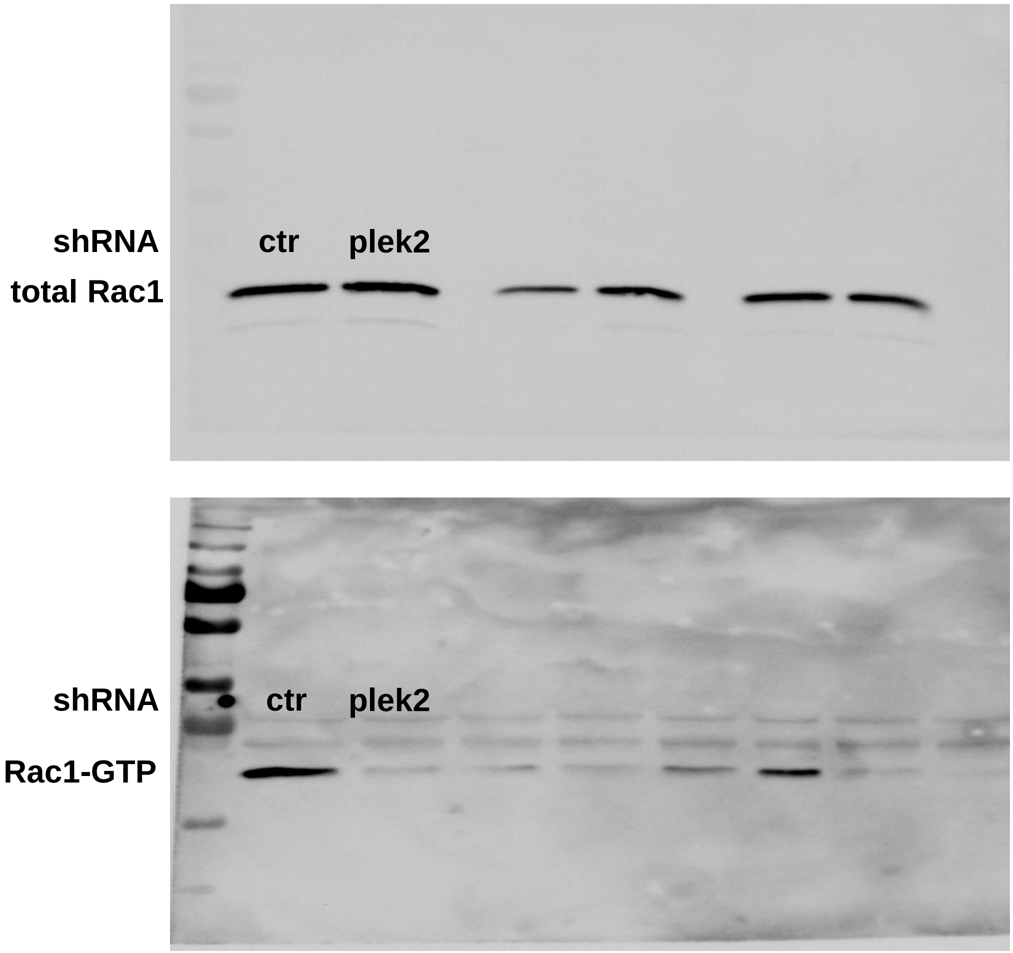

Figure 6g

shRNA ctr plek2
plek2

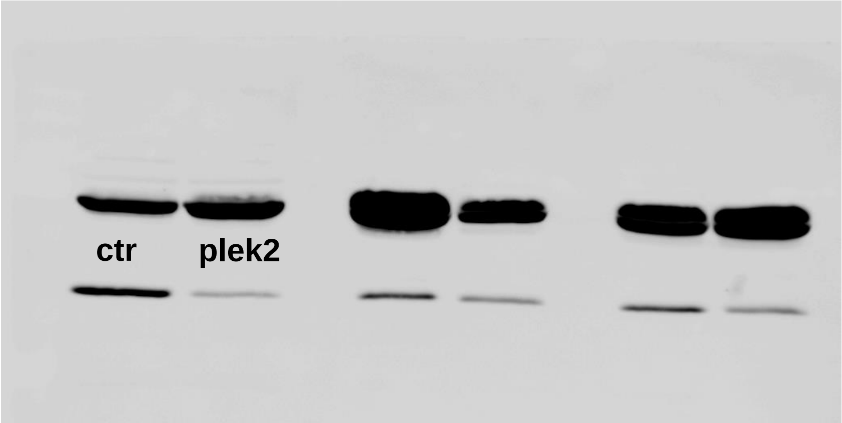

shRNA ctr plek2
β-actin

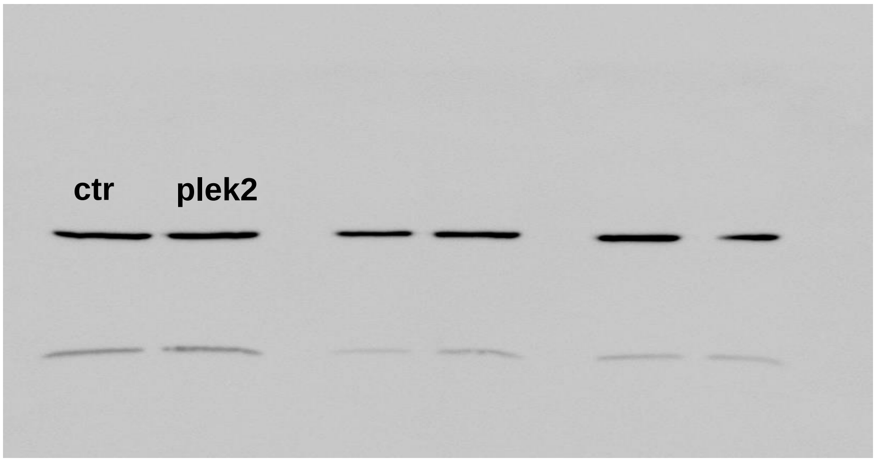

Supplementary Figure 4a

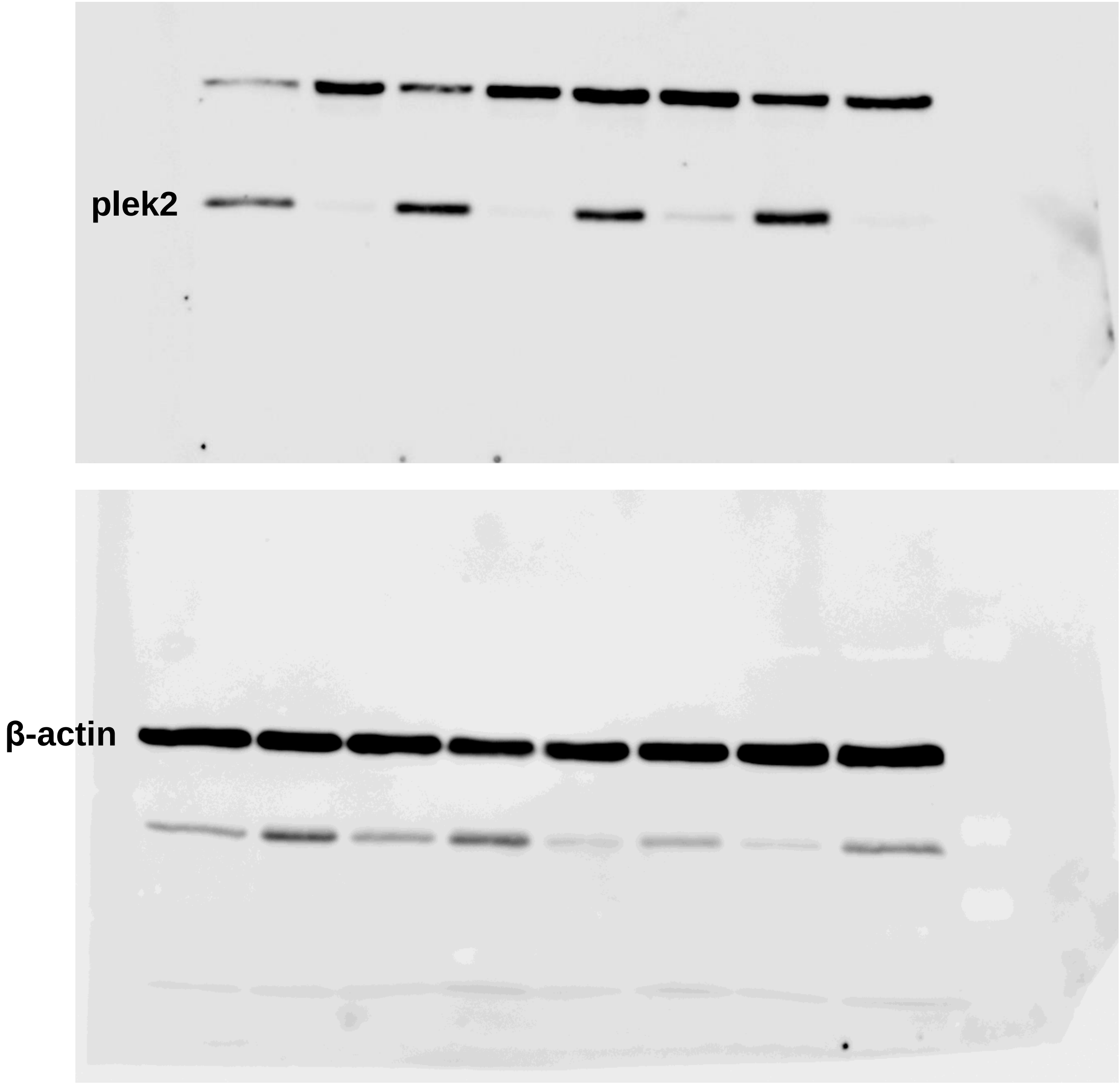

Supplementary Figure 4c

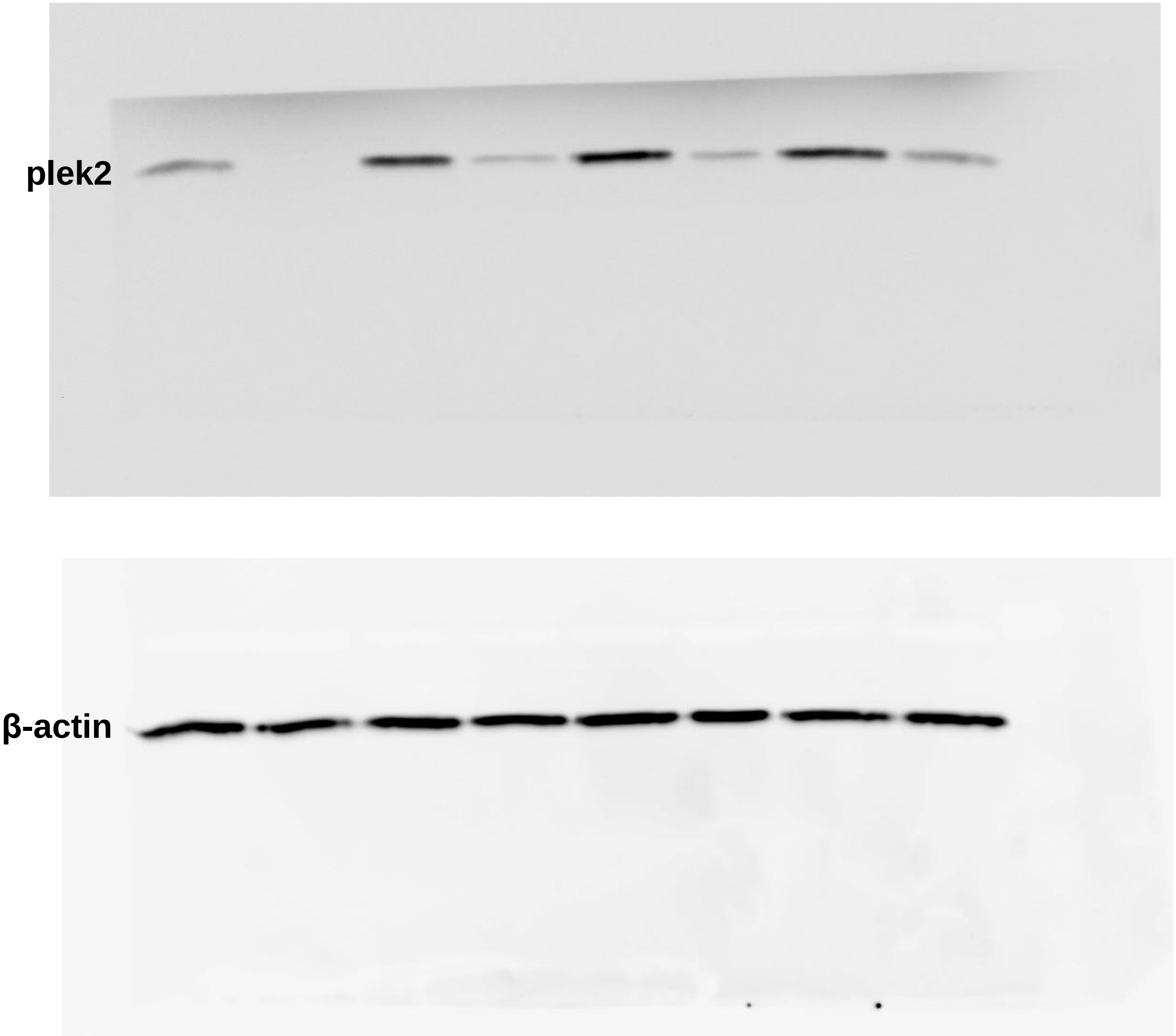

SUPPLEMENTAL METHODS

Mouse phlebotomy: Phlebotomy was performed via retro-orbital puncture with an average total of 0.4 mL of blood removed over two days; mice were sacrificed 24 hours after the second phlebotomy.

PCR: Primers used for the PCR are: plek2-forward AAAGACCTTTCTGGGCTCCT, plek2-reverse CCCTACTGGCCTGAGAAAGT; FasL-forward ACCGGTGGTATTTTTCATGG, FasL-reverse TTTAAGGCTTTGGTTGGTGA; Fam132b-forward ATGGGGCTGGAGAACAGC, Fam132b-reverse TGGCATTGTCCAAGAAGACA. Cycle threshold (Ct) values were calculated by the CFX Manager™ Software. Relative gene expression levels were expressed as the difference in Ct values (ΔCt) of the target gene and the housekeeping gene GAPDH as described (1) in each sample and normalized to GAPDH expression. All PCRs were performed in triplicate.

RNA seq: Total RNA was extracted from freshly sorted bone marrow Pro-E as above and stored at -80C until processing. The library preparation was performed using polyA+ enriched RNA according to the manufacturer's instructions (Illumina) and then sequenced on a Solexa sequencing cell. Reads were aligned using STAR against mm10 with –outFilterMultimapNmax 1 option. Differential expression analysis and FPKM levels were calculated using Homer and EdgeR using all 3 replicates for each experiment, counting reads within exons on both strands at the gene level. AnalyzeRepeats.pl rna mm10 -strand both -count exons –condenseGenes Average FPKM values were calculated for each category using all 3 replicates for each group of sorted Pro-E.

Western Blot: Samples were separated by sodium dodecyl sulfate–polyacrylamide gel electrophoresis (SDS-PAGE) using 10-12% polyacrylamide gels and transferred to

nitrocellulose membrane (Biorad). Membranes were blocked for 1 hour in TBST (10 mMTris-HCl, pH 8.0; 150 mM NaCl; 0.05% Tween 20) containing 5% skim milk or 5% BSA, followed by overnight incubation with commercially available primary antibodies (anti-plek2 1/1000 (Proteintech); anti-cofilin 1/1000 (Santa Cruz); anti-Rac1 1/1000 (Cell Signaling Technology); anti-VDAC1 1/1000 (Millipore); anti-cytochrome C 1/1000 (abcam); and anti-GAPDH 1/4000 (Invitrogen)). Blots were washed and incubated for 1 hour at room temperature (RT) with the secondary antibody (horseradish peroxidase [HRP] conjugated (Thermo Scientific)). Immunoreactive bands were visualized by the enhanced chemiluminescence (ECL) method (Amersham Bioscience) according to standard procedures.

<u>Cell culture experiments</u>: fetal liver cells were seeded (at cell density of 3 x 10^5 cells / mL) in fibronectin-coated wells (BD Discovery Labware, Bedford, MA) and directly treated with different concentrations of NSC23766 (Sigma, SML0952 (2,3,4)) or ROS scavenger N-acetyl-cysteine (NAC, Sigma, A7250), to specifically inhibit Rac1 and ROS in early stage of erythropoiesis. In brief, to evaluate effects on early stages of erythropoiesis, purified cells were cultured for 12 hours in Epo-free media with stem cell factor (SCF), interleukin-6 and FTL3 ligand (SCF media) to maintain their progenitor status, maintain cell survival and allow the expression of the infected shRNA. The cells were then transferred to Epo-containing media (Iscove's modified Dulbecco's medium (IMDM) containing 15% FBS, 1% detoxified BSA (Sigma-Aldrich), 200ug/mL holo-transferrin (Sigma), 10 ug/mL recombinant human insulin (Sigma-Aldrich), 2mM L-glutamine, 10-4 M mercaptoethanol, and 2U/mL recombinant human Epo (Amgen)), and continually cultured for 2 days. To study late stage terminal erythropoiesis, cells were immediately cultured in Epo media after infection. Samples were collected at 24 and 48 hours after resuspension in Epo-containing media for proliferation and enucleation analysis by flow cytometry, and for Rac pulldown.

Immunofluorescence: 2-3x10^5 bone marrow cells were seeded on glass coverslips coated with poly-L-lysine (Corning BioCoat™12 mm diameter) in 24-well plates, fixed with 4% paraformaldehyde in PBS, pH 7.2 at RT for 20 minutes, and permeabilized with ice-cold 0.1% Triton X-100 in PBS for 10 min. For mitochondrial staining, cells were resuspended in pre-warmed staining solution containing the MitoTracker probe (Invitrogen) before permeabilization/fixation. Cells were then stained with rabbit anti-plek2 (1:100 (Proteintech)), mouse anti-cofilin (1:250 (Santa Cruz)) antibodies overnight at 4C. After washing in PBS, Alexa Fluor®488 goat anti-rabbit IgG (1:500, Invitrogen), Alexa Fluor®647 donkey-anti-mouse IgG (1:500 (Jackson Immunoresearch)) were added for 1 hour at RT. Nuclei were identified using DAPI-Antifade (Molecular Probes). For analyses, for each high-power field, regions of interest (ROI) were defined by creating binary image masks through automatic thresholding of DAPI and plek2 positive staining. Noise was filtered out by applying a median filter (3 × 3 pixel radius). A cytoplasmic ROI was created by subtracting the DAPI staining mask (nuclear ROI) from the plek2 mask, and each of these ROI masks were applied, to the original plek2 staining images to separate nuclear and cytoplasmic staining within each high power field. Data were exported from ImageJ and used to generate histograms into Microsoft Excel software.
Nuclear:cytoplasmic ratio was used as a relative measure of plek2 nuclear localization. Nuclear and cytoplasmic histogram data were first normalized for the total number of cells/nuclei included in the analysis and then comparison was made of the average of staining intensities. For cofilin expression analysis, each high field was quantified for the mean fluorescence intensity and normalized for the total number of cells/nuclei by ImageJ software.

For mitochondrial cofilin co-localization, for each high power field, ROI were defined by creating binary image masks through thresholding. Each of these ROI masks were then compared, by the image calculator, and subjected to a 3D object counter to obtain the overlap volumes (volumes shared by cofilin and mitochondrial positive staining) by using ImageJ software.

Proximity Ligation Assay: 3x10^5 single stages of sorted bone marrow cells were seeded on glass coverslips coated with poly-L-lysine (12 mm diameter) in 24-well plates and fixed with 4% PFA in PBS, pH 7.2 at RT for 20 min, permeabilized with ice-cold 0.1% Triton X-100 in PBS for 10 min. Two primary antibodies selected from two different host species (rabbit anti-plek2, 1/100 (Proteintech); mouse anti-Rac1, 1/250 (BD Bioscience)) are used together with the generic Duolink species-specific secondary antibodies containing unique DNA strands that template the hybridization of added oligonucleotides. When in close proximity (<40 nm), the oligonucleotides are ligated by a ligase to form a circular template. This template, still anchored to the antibody, is subsequently amplified and detected using complementary labeled oligonucleotide probes (5,6,7). The resulting distinct spots were derived from single-molecule protein interaction events. Quantifications of PLA mean fluorescence intensity per cell was performed by using ImageJ software.

FRET: Mouse bone marrow erythroblasts were transfected with the biosensor expression construct, using jetPRIME DNA (0.75ug of DNA for 2.25ul of het prime for a 12 well plate) and siRNA transfection reagent (Thermofisher).The cells were plated at $2x10^5$ cells/mL density on glass coverslips coated with poly-L-lysine (Corning BioCoat™12 mm diameter) and cultured for 24 hours to allow the expression of the biosensor. Cells were then fixed in 4% PFA and imaged using a Leica DMi8 widefield microscope (Leica Microsystems Heidelberg, Manheim, Germany) equipped with a Spectra X LED light source and a monochromatic camera DFC 9000 GT (Leica, 2048 x 2048 pixels, 16bit). CFP and FRET images were acquired with a 63 x 1.40NA OIL HC PL APO objective by exciting with a 440nm LED line and using a 430/24 excitation filter, 455,515 double dichroic and 470/24 or 535/30 emission filter for CFP or FRET detection, respectively. For each image, background was subtracted in CFP and FRET channel. Then a binary mask was generated through intensity thresholding and was applied to the matched

FRET channel and CFP image. Noise was filter out with a median filter radius=10. Final FRET image were obtained by dividing FRET by CFP channel. A linear pseudo color lookup table was applied, and the ratio values were normalized to the lower scale value.

Retroviral constructs: For the generation of retroviral particles, HEK293T cells were transfected with plasmids and the retroviral packaging construct Pcl-Eco in a 2:1 ratio by TransIT-LT1 transfection reagent (Mirus) according to the manufacturer's protocols. Viral supernatants were collected after 48-72 hours. Retroviral infection was performed by resuspending purified Ter119 negative FLCs from both WT and th1/th1 mice in viral supernatants with 8 ug/mL polybrene (Sigma) and centrifuged at 1800 rpm for 1.5 hours at 37°C. After spin-infection, the viral supernatants were immediately removed and fresh media added.

Active Rac1 pulldown: Pellets from 1x10^7 FLCs from WT and thal infected with both MSCV-IRES-GFP-shRNActr and shRNAplek2, as well as CD45- bone marrow erythroblasts from WT, thal and apo-transferrin thal treated mice, were lysed with Lysis/Binding/Wash buffer on ice for 5 minutes and the supernatants transferred to a new tube. To ensure the pull-down procedures were working properly, lysates were incubated with 0.5M EDTA pH 8 and 0.1mM GTPyS (positive control) or 1mM GDP (negative control), and the mixture incubated at 30°C for 15 minutes with constant agitation. Spin cups with glutathione resin and GST-human Pak1-PBD were prepared, and the samples supernatants were transferred in to it. The reaction mixtures were incubated at 4°C for 1 hour with gentle rocking, and the resins were then washed for 3 times with 400ul of Lysis/Binding/Wash buffer. Resins were incubated 2 minutes with 50ul of 2x reducing sample buffer and the eluted samples were then heated for 5 minutes at 95-100°C and electrophoresed on a 12% acrylamide gel.

SUPPLEMENTAL FIGURE LEGENDS

Suppl Figure 1: Inhibition of ROS with low dose of ROS scavenger promotes enucleation in higher degree in early stage β-thalassemic erythroblasts. Flow cytometry statistical analysis of enucleation of Ter119 negative mouse fetal liver erythroblasts from WT and β-thalassemic cultured in Epo containing medium in the absence or presence of 5mM of NAC for 48 hours (3-4 mice/group). Hoechst was used for the DNA staining. All data are reported as means ± s.e.m and $p<0.05$ was considered statistically significant (Student's unpaired t test) (*p≤0.05, **p≤0.005, ***p≤0.0005, ****p≤0.00005). (n=3-4 mice/category) (WT = wild type; NAC = N-acetyl L cysteine).

Suppl Figure 2: Validation of RNA sequencing results using apo-transferrin as a tool to evaluate ineffective erythropoiesis and its reversal in β-thalassemia. Relative expression of Podxl **(A)** and Fam132b **(B)** in RNA sequencing analysis demonstrates significantly increased expression in th1/th1 bone marrow Pro-E, partially normalized after apoTf treatment. All data are reported as means ± s.e.m and $p<0.05$ was considered statistically significant (one-way ANOVA) (*p≤0.05, **p≤0.005, ***p≤0.0005, ****p≤0.00005). Relative expression of Plek2 **(C)**, Podxl **(D)**, and Fam132b **(E)** in RNA sequencing analysis is unaltered by apoTf treatment in WT mice. All data are reported as means ± s.e.m. and $p<0.05$ was considered statistically significant (Student's unpaired t test) (*p≤0.05, **p≤0.005, ***p≤0.0005, ****p≤0.00005). (WT = wild type, apoTf = apo-transferrin; Plek2 = pleckstrin-2; FPKM = fragments for kilobase millions)

Suppl Figure 3: Pleckstrin-2 loss results in an embryonic lethal phenotype early in erythroid development due to increased erythroblast apoptosis and impaired enucleation. (A) Timed matings of thal plek2+/- mother and plek2+/- father lead to absence of th3/+plek2-/- embryos at E11.5. Fetal liver cells from embryos at E11.5 exhibit a persistent

enucleation defect **(B)** and increased erythroblast apoptosis as measured by activated caspase 3 **(C)** in thal plek2+/- relative to thal embryos (thal = β-thalassemic; plek2 = pleckstrin-2)

<u>Suppl Figure 4</u>**: Pleckstrin-2 knockdown in later stage erythroblasts results in a smaller effect on enucleation and proliferation relative to pleckstrin-2 knockdown in earlier stage erythroblasts. (A)** Ter119 negative fetal liver cells from E13.5 WT and β-thalassemic embryos were transfected with nt-shRNA or plek2-shRNA and analyzed after 48 hours in expansion followed by Epo-containing media to enable nt-shRNA or plek2-shRNA transfection in CFU-E, early stage erythroblasts. Western blot demonstrates appropriate plek2 knockdown. **(B)** Cells cultured for 72 hours demonstrate a persistent enucleation defect in thalassemic erythroblasts with additionally decreased enucleation in plek2-shRNA transfected thalassemic erythroblasts. **(C)** Ter119 negative fetal liver cells from E13.5 WT and β-thalassemic embryos were transfected with nt-shRNA or plek2-shRNA and analyzed after 48 hours in Epo-containing media to enable nt-shRNA or plek2-shRNA transfection in Pro-E and Baso-E, later stage erythroblasts. Western blot demonstrates appropriate plek2 knockdown. **(D)** Flow cytometry analysis of *later* stage erythroblasts (Pro-E and Baso-E) isolated from fetal liver cells from E13.5 WT and β-thalassemic embryos including both nucleated (Q2 quadrant; Hoechst high (32)) and enucleated (Q4 quadrant; Hoechst low (32)) cells after nt-shRNA, and nucleated (Q6 quadrant; Hoechst high (32)) and enucleated (Q8 quadrant; Hoechst low (32)) cells after plek2-shRNA transfection. Plek2-shRNA transfection does not decrease enucleated (Q8) cells in WT but only in β-thalassemic cells with a such smaller degree of suppression relative to early stage (CFU-E) erythroblasts (Figure 3A). All data are reported as means ± s.e.m. and $p<0.05$ (one-way ANOVA) was considered statistically significant. **$p \leq 0.005$ vs. WT nt-shRNA; +$p \leq 0.05$ vs. nt-shRNA ++$p \leq 0.005$ vs. nt-shRNA. (WT= wild type; plek2 = pleckstrin-2; MFI = mean fluorescence index; GFP = green fluorescent protein).

__Suppl Figure 5__**: Pleckstrin-2 activation and membrane localization during erythroid precursor differentiation.** Statistical analysis of confocal immunofluorescence microscopy from Figure 4A for the percentage of membrane plek2 localization relative to total plek2 during terminal erythropoiesis in WT **(A)**, β-thalassemic **(B)** and apoTf-treated β-thalassemic mice **(C)**. The pattern of plek2 membrane localization is normalized by apo-transferrin in β-thalassemic erythroblasts. (WT = wild type; thal = β-thalassemic; apoTf = apo-transferrin; plek2 = pleckstrin-2; Pro-E = pro-erythroblasts; Baso-E = basophilic erythroblasts; Poly-E = polychromatophilic erythroblasts; Ortho-E = orthochromatophilic erythroblasts).

__Suppl Figure 6__**: Enhanced activation of pleckstrin-2 in erythroblasts is a physiological response during stress erythropoiesis.** Flow cytometry analysis of bone marrow erythroblast fraction **(A)**, ROS **(B)**, and Annexin V **(C)** in WT and phlebotomized WT mice demonstrates increased proliferation without increased apoptosis despite increased ROS (n=3 mice/category; 2-4 month age and gender matched). **(D)** Statistical analysis for confocal immunofluorescence microscopy of membrane pleckstrin-2 localization in bone marrow erythroblasts from WT and phlebotomized WT mice demonstrating increased membrane-associated pleckstrin-2 localization in all stages of terminal erythropoiesis. All data are reported as means ± s.e.m. and $p<0.05$ was considered statistically significant (two-way ANOVA) (*$p\leq0.05$, **$p\leq0.005$, ***$p\leq0.0005$). (WT = wild type; PhL= phlebotomized; plek2 = pleckstrin-2; ROS = reactive oxygen species; MFI = mean fluorescent index).

__Suppl Figure 7__**: Cofilin expression and pleckstrin-2:cofilin interaction is unchanged in late stage β-thalassemic erythroblasts. (A)** Confocal immunofluorescence microscopy of cofilin expression in Ortho-E stage from sorted (2-3 mice pooled per data point, n=6 data points per category) WT, β-thalassemic and apoTf-treated β-thalassemic erythroblasts. The confocal images were representative cells from at least 50 cells analyzed in each condition in 6 biological

replicates from 2 different experiments. Scale bar 5µm. **(B)** MFI of cofilin expression displayed in (A) demonstrates no differences in Ortho-Es between WT and β-thalassemic, but is decreased in apoTf treated β-thalassemic mice. **(C)** Confocal immunofluorescence microscopy of plek2 and cofilin co-localization in Ortho-E stage of sorted (2-3 mice pooled per data point, n=3 data points per category) WT, β-thalassemic and apoTf-treated β-thalassemic erythroblasts. The confocal images were representative optical sections of the cell from at least 50 cells in each condition in 2 different experiments. Scale bar 5µm. **(D)** MFI of plek2:cofilin co-localizations in Ortho-E in WT, β-thalassemic and apoTf-treated β-thalassemic mice demonstrates no differences between WT, β-thalassemic and after apoTf treatment. All data are reported as means ± s.e.m. and $p<0.05$ (one-way ANOVA) was considered statistically significant. (*$p \leq 0.05$, **$p \leq 0.005$, ***$p \leq 0.0005$). (WT = wild type; thal = β-thalassemic; apoTf = apo-transferrin; plek2 = pleckstrin-2; ROS = reactive oxygen species; MFI = mean fluorescence index; Pro-E = pro-erythroblasts; Baso-E = basophilic erythroblasts; Poly-E = polychromatophilic erythroblasts; Ortho-E = orthochromatophilic erythroblasts).

<u>Suppl Figure 8</u>: Pleckstrin-2:RacGTPase interaction and Rac1 activation are not increased in late stage β-thalassemic erythroblasts. (A) Confocal immunofluorescence microscopy of Rac1 and plek2 interaction (red) using the proximity ligation assay in sorted (2-3 mice pooled per data point, n=3 data points per category) Ortho-E from WT, β-thalassemic, and apoTf-treated β-thalassemic mice. Scale bar 5µm. **(B)** Statistical analysis of confocal immunofluorescence microscopy of Rac1 and plek2 interaction using the proximity ligation assay quantifying Rac1 and plek2 interaction, demonstrating no differences in interaction in late (i.e. Ortho-E) stage β-thalassemic erythroblasts relative to WT, and after apoTf treatment. All data are reported as means ± s.e.m. and $p<0.05$ was considered statistically significant (one-way ANOVA) (*$p \leq 0.05$, **$p \leq 0.005$, ***$p \leq 0.0005$). (WT = wild type; apoTf = apo-transferrin; MFI = mean fluorescence intensity). **(C)** Fetal liver cells in culture demonstrate not statistical increased

activated Rac1 in β-thalassemic erythroblasts relative to WT controls in late erythropoiesis. All data are reported as means ± s.e.m. and $p<0.05$ was considered statistically significant (Student's unpaired t test) (*p≤0.05, **p≤0.005, ***p≤0.0005), (n=3 mice/category) (WT = wild type; thal = β-thalassemic; apoTf = apo-transferrin; MFI = mean fluorescence intensity).

Suppl Figure 9: RacGTPase inhibitor blocks enucleation in erythroid precursors. Analyzed by flow cytometry, the enucleated fraction is suppressed with escalating doses of Rac-specific inhibitor NSC23766 in both WT and β-thalassemic fetal liver cells (4-5 mice/group). All data are reported as means ± s.e.m. and $p<0.05$ was considered statistically significant (ANOVA) (**p≤0.005, ***p≤0.0005). (WT = wild type; thal = β-thalassemic).

Suppl Figure 10: Full blots from Figures 1F, 5A, 5G, 6A, and 6G, as well as Suppl Figures 4A and 4C

REFERENCES

1. Pfaffl MW. A new mathematical model for relative quantification in real-time RT-PCR. Nucleic Acids Res. 2001;29(9):e45.
2. Ji P, Jayapal SR, Lodish HF. Enucleation of cultured mouse fetal erythroblasts requires Rac GTPases and mDia2. Nat Cell Biol. 2008;10(3):314-321.
3. Nassar, N., Cancelas, J., Zheng, J., Williams, D. A. & Zheng, Y. Structure-function based design of small molecule inhibitors targeting Rho family GTPases. Curr. Top. Med. Chem. 2006;6:1109–1116.
4. Akbar, H., Cancelas, J., Williams, D. A., Zheng, J. & Zheng, Y. Rational design and applications of a Rac GTPase-specific small molecule inhibitor. Methods Enzymol. 2006;406:554–565.
5. Söderberg O, Gullberg M, Jarvius M, Ridderstråle K, Leuchowius KJ, Jarvius J, Wester K, Hydbring P, Bahram F, Larsson LG, Landegren U. Direct observation of individual endogenous protein complexes in situ by proximity ligation. Nat Methods. 2006;3(12):995-1000.
6. Jarvius M, Paulsson J, Weibrecht I, Leuchowius KJ, Andersson AC, Wählby C, Gullberg M, Botling J, Sjöblom T, Markova B, Ostman A, Landegren U, Söderberg O. In situ detection of phosphorylated platelet-derived growth factor receptor beta using a generalized proximity ligation method. Mol Cell Proteomics. 2007;6(9):1500-9.
7. Fredriksson S, Gullberg M, Jarvius J, et al. Protein detection using proximity-dependent DNA ligation assays. Nat Biotechnol. 2002;20(5):473-477.
