# Supplementary material for: Pleckstrin-2 is essential for erythropoiesis in β-thalassemic mice, reducing apoptosis and enhancing enucleation
Source: Commun Biol. 2021 May 3;4:517. doi: 10.1038/s42003-021-02046-9 (PMC8093212; doi:10.1038/s42003-021-02046-9)
Supplement: Supplementary file 3 — Reporting summary. [file 42003_2021_2046_MOESM3_ESM.pdf]

nature research

Corresponding author(s): Yelena Ginzburg

Last updated by author(s): Mar 1, 2021

Reporting Summary

Nature Research wishes to improve the reproducibility of the work that we publish. This form provides structure for consistency and transparency in reporting. For further information on Nature Research policies, see our Editorial Policies and the Editorial Policy Checklist.

Statistics

For all statistical analyses, confirm that the following items are present in the figure legend, table legend, main text, or Methods section.

n/a	Confirmed	
☐	☒	The exact sample size (n) for each experimental group/condition, given as a discrete number and unit of measurement
☐	☒	A statement on whether measurements were taken from distinct samples or whether the same sample was measured repeatedly
☐	☒	The statistical test(s) used AND whether they are one- or two-sided<br>*Only common tests should be described solely by name; describe more complex techniques in the Methods section.*
☐	☒	A description of all covariates tested
☒	☐	A description of any assumptions or corrections, such as tests of normality and adjustment for multiple comparisons
☐	☒	A full description of the statistical parameters including central tendency (e.g. means) or other basic estimates (e.g. regression coefficient) AND variation (e.g. standard deviation) or associated estimates of uncertainty (e.g. confidence intervals)
☐	☒	For null hypothesis testing, the test statistic (e.g. F, t, r) with confidence intervals, effect sizes, degrees of freedom and P value noted<br>*Give P values as exact values whenever suitable.*
☒	☐	For Bayesian analysis, information on the choice of priors and Markov chain Monte Carlo settings
☒	☐	For hierarchical and complex designs, identification of the appropriate level for tests and full reporting of outcomes
☒	☐	Estimates of effect sizes (e.g. Cohen's d, Pearson's r), indicating how they were calculated

Our web collection on statistics for biologists contains articles on many of the points above.

Software and code

Policy information about availability of computer code

Data collection	none
Data analysis	none

For manuscripts utilizing custom algorithms or software that are central to the research but not yet described in published literature, software must be made available to editors and reviewers. We strongly encourage code deposition in a community repository (e.g. GitHub). See the Nature Research guidelines for submitting code & software for further information.

Data

Policy information about availability of data

All manuscripts must include a data availability statement. This statement should provide the following information, where applicable:

- Accession codes, unique identifiers, or web links for publicly available datasets
- A list of figures that have associated raw data
- A description of any restrictions on data availability

Figure 1-6 all contain original data for which associated raw data is provided in Supplementary Data 1

Field-specific reporting

Life sciences study design

All studies must disclose on these points even when the disclosure is negative.

Sample size	biological variants, a minimum of n=3 per group, was used in all data sets.
Data exclusions	none
Replication	all experiments were repeated at least twice to confirm findings
Randomization	NA
Blinding	NA

Reporting for specific materials, systems and methods

We require information from authors about some types of materials, experimental systems and methods used in many studies. Here, indicate whether each material, system or method listed is relevant to your study. If you are not sure if a list item applies to your research, read the appropriate section before selecting a response.

Materials & experimental systems

n/a	Involved in the study	
☐	☒	Antibodies
☐	☒	Eukaryotic cell lines
☒	☐	Palaeontology and archaeology
☐	☒	Animals and other organisms
☒	☐	Human research participants
☒	☐	Clinical data
☒	☐	Dual use research of concern

Methods

n/a	Involved in the study	
☒	☐	ChIP-seq
☐	☒	Flow cytometry
☒	☐	MRI-based neuroimaging

Antibodies

Antibodies used	commercially available for analysis of biologically-relevant endpoints, including:<br>FOR FLOW CYTOMETRY: anti-mouse TER119-phycoerythrin Cy7 (PE-Cy7) (BioLegend, 116222), CD44-allophycocyanin (APC) (Tonbo, Biosciences, 20-0441-U100), and CD71-PE (BD Pharmigen, 553267)<br>FOR WESTERN BLOT: anti-plek2 1:1000 (Proteintech, 11685-1-AP); anti-cofilin 1:1000 (Santa Cruz, sc-376476); anti-Rac1 1:1000 (Cell Signaling Technology, 2465); anti-VDAC1 1:1000 (Millipore, MABN504); anti-cytochrome C 1:1000 (abcam, ab90529); and anti-GAPDH 1:4000 (Invitrogen)<br>FOR IMMUNOFLUORESCENCE: rabbit anti-plek2 (1:100 (Proteintech)), mouse anti-cofilin (1:250 (Santa Cruz))<br>FOR PLA: rabbit anti-plek2, 1:100 (Proteintech); mouse anti-Rac1, 1:250 (BD Bioscience, 610650)
Validation	all validations were performed commercially and proper positive and negative controls were included in the initial use of the reagents.

Eukaryotic cell lines

Policy information about cell lines

Cell line source(s)	ATCC® CRL-3216™
Authentication	the cell line was not authenticated
Mycoplasma contamination	cells were not tested for mycoplasma contamination
Commonly misidentified lines<br>(See ICLAC register)	None

Animals and other organisms

Policy information about studies involving animals; ARRIVE guidelines recommended for reporting animal research

Laboratory animals	mouse, M/F, C57BL6 background, 3-4 month old age and gender matched. All studies were approved by the Institutional Animal Care and Use Committee.
Wild animals	no wild animals involved

Field-collected samples	no field-collected samples
Ethics oversight	all animal related work was approved by IACUC

Note that full information on the approval of the study protocol must also be provided in the manuscript.

Flow Cytometry

Plots

Confirm that:

- [x] The axis labels state the marker and fluorochrome used (e.g. CD4-FITC).
- [x] The axis scales are clearly visible. Include numbers along axes only for bottom left plot of group (a 'group' is an analysis of identical markers).
- [x] All plots are contour plots with outliers or pseudocolor plots.
- [x] A numerical value for number of cells or percentage (with statistics) is provided.

Methodology

Sample preparation	bone marrow was flushed directly from femura at time of sacrifice. samples were first washed, bead separated, and counted prior to staining with antibodies and flow analysis or sorting.
Instrument	Samples were analyzed using either FACSCanto I or LSRFortessa flow cytometer (BD Biosciences). Cell sorting was performed on a CSML4Cell Sorter (BD Biosciences) using DIVA Software.
Software	DIVA software
Cell population abundance	erythroblast represent 66-95% of bone marrow cells
Gating strategy	all nucleated cells were identified, doublets and dump-channel positive cells were excluded, and marker positive cells (TER119, CD44, and CD71) were acquired both for analysis and sorting. backgating, cytospin, and microscopy confirmed the proper collection of cells of interest.

- [x] Tick this box to confirm that a figure exemplifying the gating strategy is provided in the Supplementary Information.
