# Supplementary material for: Pleckstrin-2 is essential for erythropoiesis in β-thalassemic mice, reducing apoptosis and enhancing enucleation
Source: Commun Biol. 2021 May 3;4:517. doi: 10.1038/s42003-021-02046-9 (PMC8093212; doi:10.1038/s42003-021-02046-9)
Supplement: Supplementary file 4 — Description of Additional Supplementary Files. [file 42003_2021_2046_MOESM4_ESM.pdf]

Description of Additional Supplementary Files

File name: Supplementary Data 1

Description: Source Data for all Main and Supplementary Figures.
